# Interface‐Confined Cavitation at an MXene/TiO_2_ Schottky Heterointerface for Non‐Thermal Juice Preservation

**DOI:** 10.1002/advs.78536

**Published:** 2026-10-28

**Authors:** Xiaoge Wu, Yunxiao Zhu, Nan Qin, Zhaoli Zhang, Qi Zhang, Yang Tao

**Affiliations:** ^1^ Key Laboratory of Arable Land Quality Monitoring and Evaluation (Yangzhou University) Ministry of Agriculture and Rural Affairs School of Environmental Science and Engineering Yangzhou University Yangzhou China; ^2^ Key Laboratory of Chinese Cuisine Intangible Cultural Heritage Technology Inheritance College of Tourism and Culinary Science Yangzhou University Yangzhou China; ^3^ School of Computer and Electronic Information Nanjing Normal University Nanjing China; ^4^ College of Food Science and Technology Nanjing Agricultural University Nanjing Jiangsu China; ^5^ Food Safety and Nutrition Experimental Teaching Demonstration Center College of Food Science and Engineering Xinjiang Institute of Technology Aksu Xinjiang China

**Keywords:** food safety, free radicals, inactivation, mechanical force, non‐thermal processing, sonochemistry

## Abstract

Non‐thermal food preservation demands catalytic platforms that achieve potent antimicrobial efficacy without compromising heat‐labile nutrients. Yet whether a sonocatalyst merely seeds bubble nucleation or actively channels transiently concentrated acoustic energy into chemical work has remained unresolved, and the violent, opaque nature of cavitation further precludes operando observation of catalyst surfaces. Herein, an MXene/TiO_2_ Schottky heterointerface is engineered with the aim of localizing cavitation collapse at the catalyst‐liquid boundary through three cooperatively acting determinants: a built‐in electric field for charge separation, hierarchical surface roughness for energy focusing, and pre‐existing oxygen vacancies for dynamic defect modulation. A convergent multi‐proxy evidence chain, comprising passive cavitation imaging, single‐bubble computational fluid dynamics, chronoamperometry, quasi‐in situ spectroscopy, and ab initio molecular dynamics (AIMD), supports a working model of this interface‐confined cavitation regime. Under 40 kHz ultrasound without external heating, the interface delivers 3‐log inactivation of the tested indicator bacteria (*E. coli* and *S. aureus*) at the laboratory scale, while preserving 82% of vitamin C and 88% of total anthocyanin content in juice. This work thus proposes interface‐confined cavitation as a transferable design rationale for sonocatalytic interfaces, with operando validation of the active interface as the decisive next step.

## Graphical abstract

This work establishes an MXene/TiO_2_ Schottky heterointerface for non‐thermal sonocatalysis. Interface‐confined cavitation and dynamic Ti^3^^+^/V₀ shuttles drive charge transport and reactive oxygen species (ROS) generation. The platform achieves 3‐log pathogen reduction while retaining heat‐labile nutrients in complex food matrices, redefining sonocatalyst design.

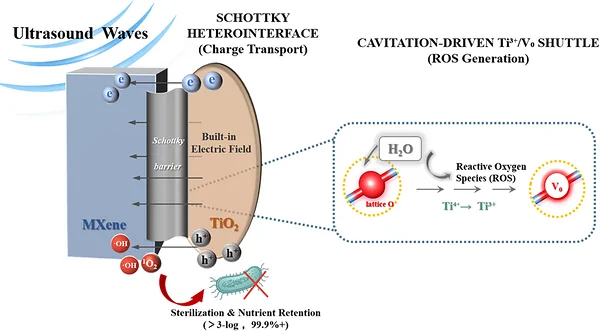

## 1 Introduction

Ensuring global food security while minimizing post‐harvest losses remains a central pillar of the United Nations Sustainable Development Goals, particularly SDG 2 (Zero Hunger) and SDG 12 (Responsible Consumption and Production) [1]. Conventional thermal pasteurization and sterilization, despite their reliable microbial inactivation efficacy, invariably compromise heat‐labile nutrients and sensory quality, creating an inherent safety–quality trade‐off that limits their applicability to high‐value liquid foods [2]. There is a pressing demand for non‐thermal technologies that achieve equivalent microbial reduction without collateral thermal damage.

Power ultrasound has emerged as a leading non‐thermal platform, inactivating microorganisms through acoustic cavitation without substantial bulk heating [3]. The chemical basis of sonochemistry rests on cavitation‐collapse‐generated hotspots that drive water dissociation into reactive radical species [4]. These foundations motivate sonodynamic strategies that integrate sonosensitizers to harvest acoustic energy and convert it into cytotoxic reactive oxygen species (ROS) [4, 5]. Yet the very intensity that makes cavitation attractive also raises a question that has defined the field and remains unanswered: where the transient acoustic energy is ultimately deposited and converted into chemical work, and whether the catalyst participates in that fate or merely seeds the nucleation event.

Titanium dioxide (TiO_2_), a prototypical sonosensitizer, has been widely studied for its chemical stability, low cost, and well‐documented physicochemical properties [6]. Yet conventional TiO_2_ suffers from rapid electron–hole recombination, a limitation broadly shared with heterojunction photocatalysts requiring interfacial engineering to suppress charge recombination [7]. Defect engineering offers a route forward, but oxygen‐vacancy‐induced phase transformations can undermine structural durability beyond an optimum defect density [8], and analogous lattice‐distortion penalties have been documented in TiO_2_‐based systems [9]. Along these lines, MXene‐derived TiO_2_ nanocomposites constructed via crystal‐defect engineering have emerged as promising hybrid catalysts [10].

The MXene (two‐dimensional transition metal carbides, nitrides, and carbonitrides)/TiO_2_ pairing draws strength from two complementary literatures that converge at a single, unresolved point: the spatial fate of cavitation energy. On one hand, MXene‐derived TiO_2_/Ti_3_C_2_ integrated heterostructures have been fabricated and evaluated for pollutant degradation [11], resting on two‐dimensional Ti_3_C_2_ nanosheets that combine high electron conductivity with tunable surface chemistry [12]. On the other hand, heterogeneous sonocatalysis reviews establish that solid particles act as cavitation nuclei at solid–liquid interfaces [13], and molecular dynamics studies show that surface roughness and wettability modulate ultrasonic cavitation behavior [14, 15, 16]. Together, these lines suggest the untested possibility that an MXene/TiO_2_ Schottky heterointerface could spatially couple cavitation energy to the catalytic interface, rather than dissipating it into the bulk, which, if realized, would turn the catalyst from a passive bystander into the active locus of chemical conversion.

In addition, methodological barriers have also constrained sonochemistry relative to its photochemical and electrochemical counterparts, while cavitation and the surface probes of materials science are fundamentally incompatible. The violent, optically opaque, acoustically driven liquid in which cavitation occurs is largely inaccessible to the ultrahigh‐vacuum and beam‐based spectroscopies needed to observe a catalyst surface during an active cavitation event. Consequently, the interfacial states that would mediate sonocatalysis, namely charge separation across a heterojunction and the acoustic‐driven evolution of defect sites, have remained black boxes, necessarily inferred from quasi‐in situ evidence rather than observed operando. Closing this gap is precisely what distinguishes an interfacial‐engineering approach, and it motivates both the present design and its evidentiary strategy. Answering this question would mark a conceptual step beyond the prevailing bulk‐radical paradigm, toward an interfacial‐engineering framework in which a designed catalyst governs both where and how acoustic energy is converted. This distinction matters acutely in real food matrices. Prior TiO_2_‐based decontamination has relied predominantly on photocatalysis and achieved far below 1‐log reduction [17], whereas the interfacial regime described here targets the route that may dominate in juice under ultrasound.

To this end, a Schottky heterointerface engineered from MXene/TiO_2_ is presented, proposing the concept of interface‐confined cavitation. The design synergistically integrates three determinants: a built‐in electric field for charge separation, hydrophilic roughness for energy focusing, and pre‐existing oxygen vacancies for dynamic defect modulation. A convergent, multi‐proxy evidence chain, drawing on cavitation imaging, electrochemistry, quasi‐in situ spectroscopy, and ab initio molecular dynamics (AIMD), supports the interface‐confined regime while explicitly delimiting what remains open to operando validation. Under purely ultrasonic driving, this interface achieves a 3‐log reduction of the tested indicator bacteria (*E. coli* and *S. aureus*) in complex juice matrices at the laboratory scale while preserving heat‐labile nutrients (82% vitamin C, 88% total anthocyanin content in blueberry juice). The evidence assembled here is consistent with a working model in which the catalyst is not merely a passive nucleation seed but also modulates where acoustic energy is deposited and how it is subsequently converted. This work offers a transferable design principle for sonocatalytic interfaces.

## 2 Results and Discussion

Figure 1 presents structural evidence for the MXene/TiO_2_ composite. Transmission electron microscopy (TEM) images (Figure 1a) reveal intimate interfacial contact between two‐dimensional MXene nanosheets and uniformly dispersed TiO_2_ nanoparticles. High‐resolution TEM (HRTEM) and the corresponding fast Fourier transform (FFT) pattern (Figure 1b) confirm atomic‐scale integration, displaying distinct lattice fringes of crystalline anatase TiO_2_ (the (101) plane) in direct contact with the layered MXene structure. High‐resolution X‐ray photoelectron spectroscopy (XPS) spectra of the Ti 2p region (Figure 1c) corroborate the coexistence of Ti^4^^+^ and reduced Ti^3^^+^ states. Electron paramagnetic resonance (EPR) spectroscopy (Figure 1d) reveals a distinct paramagnetic signal at *g* = 2.003 in the composite, conventionally assigned to oxygen‐vacancy‐related defect states and/or Ti^3^^+^ trapped‐electron centers within the TiO_2_ lattice [8, 18].

**FIGURE 1:**
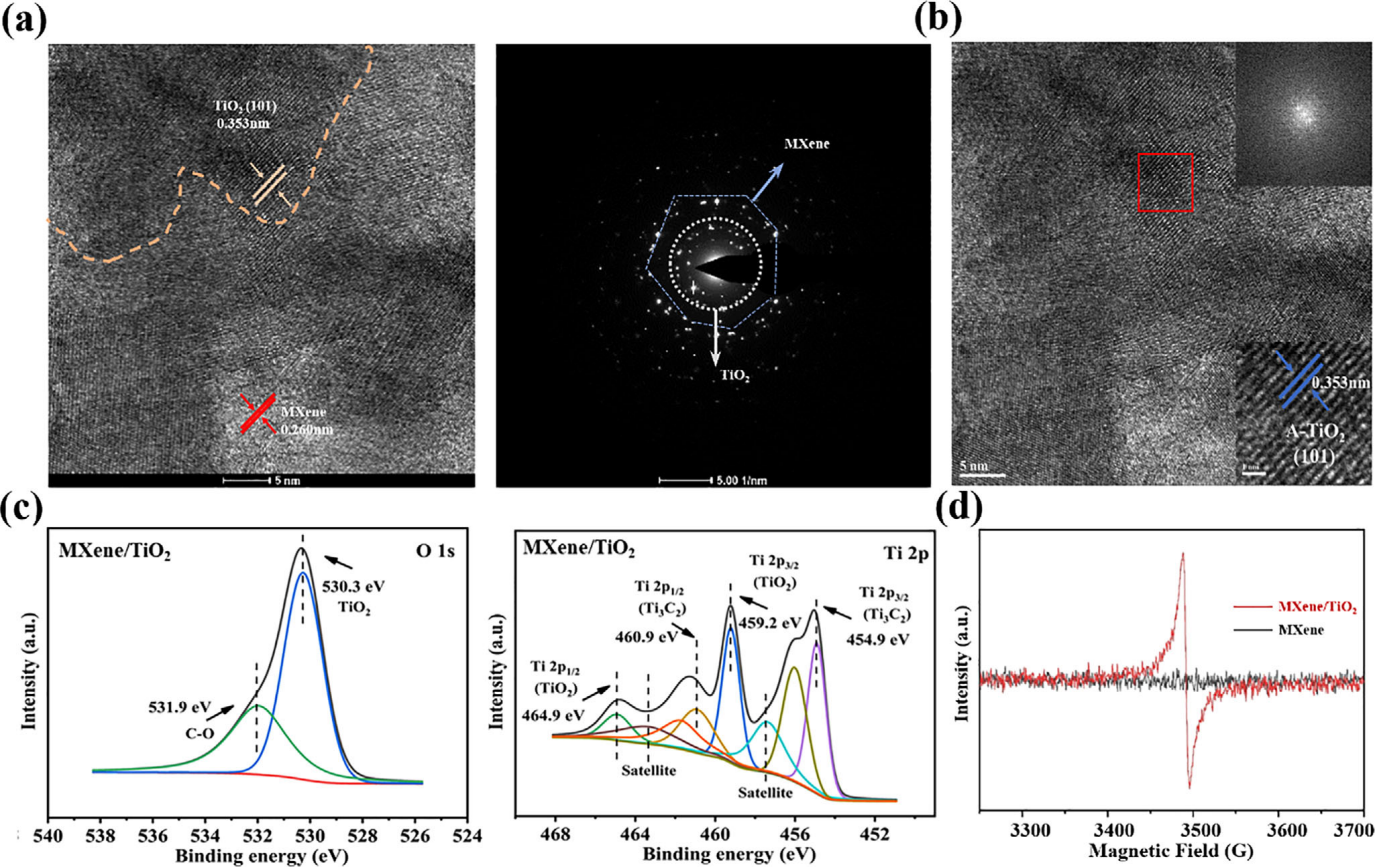
Structural Characterization and Surface Chemical States of the MXene/TiO_2_ Composite. (a) TEM image showing the intimate interfacial contact between TiO_2_ nanocrystals and the MXene support. The relatively low contrast of MXene is due to the partial delamination and thinning of the nanosheets, while the crystalline structure is verified by the selected‐area electron diffraction (SAED) pattern (inset). (b) High‐resolution TEM (HRTEM) image of the TiO_2_ (101) facet in direct contact with MXene, with the inset showing the corresponding fast Fourier transform (FFT) pattern. (c) XPS spectra of the Ti 2p and O 1s regions. (d) Electron paramagnetic resonance (EPR) spectrum exhibiting a paramagnetic signal at *g* = 2.003, consistent with oxygen‐vacancy‐related defect states and/or Ti^3^^+^ trapped‐electron centers.

Complementary characterizations (Figures S1 and S2) further confirm the material structure. Field‐emission scanning electron microscopy (FE‐SEM) images (Figure S1a) show the preserved layered, accordion‐like morphology of the HF‐etched MXene substrate [12], upon which TiO_2_ nanocrystals are homogeneously anchored (Figure S2). X‐ray diffraction (XRD) patterns (Figure S1b) confirm the formation of the composite: the characteristic (002) peak of MXene is retained albeit with reduced intensity and a slight low‐angle shift, consistent with intercalation of TiO_2_ precursors or solvent molecules and partial delamination of the MXene nanosheets during synthesis [12]. Meanwhile, new diffraction peaks corresponding to the anatase phase of TiO_2_ emerge and sharpen. Raman spectroscopy (Figure S1c) further supports the coexistence of both components, displaying the characteristic D and G bands of the MXene carbon structure [19] alongside the Raman‐active modes of anatase TiO_2_ [20]. The moderate I_D_/I_G_ ratio indicates a certain level of structural disorder, while the robust G band suggests that the MXene carbon lattice remains largely intact, a condition that may be favorable for maintaining structural stability under ultrasonic cavitation. N_2_ physisorption analysis (Figure S3a) reveals a specific surface area of 8.81 m^2^ g^−^^1^ for the MXene/TiO_2_ composite. The physical significance of the surface area lies chiefly in providing accessible surface‐active sites and a stable interfacial charge‐separation platform, rather than in maximizing adsorption capacity. This interpretation aligns with the interface‐dominated mechanism reflected by the DFT, electrochemical, and cavitation‐mapping results presented below and is further supported by precedents in which heterostructure catalytic activity does not scale linearly with Brunauer–Emmett–Teller (BET) surface area.

Density functional theory (DFT) calculations (Figure S4) provide a structural framework for interpreting the observed behavior. The calculations reveal that the projected surface Ti‐3d d‐band center, referenced to the Fermi level, upshifts from −1.52 eV in pristine TiO_2_ to −0.38 eV in Ti_3_C_2_, and reaches −0.31 eV at the TiO_2_/Ti_3_C_2_ heterointerface (Figure S4c). This upshift places the d‐electron energy levels of the interfacial Ti sites closer to the Fermi level, theoretically associated with enhanced electron participation in surface reactions and in line with the facilitated charge transfer across the heterointerface observed experimentally [21]. This interpretation accords with the electrochemical measurements. The electrochemical impedance spectroscopy (EIS) Nyquist plot (Figure S5a) exhibits a smaller arc radius for the MXene/TiO_2_ composite compared to pristine TiO_2_, indicating a reduced interfacial charge‐transfer resistance (R_ct_) at the heterointerface. Meanwhile, the Mott–Schottky plot (Figure S5b) shows a negative shift in the flat‐band potential for the composite relative to pristine TiO_2_, suggesting an upward bending of the energy bands at the Schottky interface that favors electron depletion and charge separation. The proposed band alignment and interfacial charge‐transfer pathway are schematically summarized in Figure S5d. This behavior is consistent with the broader principle of heterojunction engineering, in which interfacial coupling between materials with complementary electronic structures facilitates charge migration and suppresses electron–hole recombination, as demonstrated in Fe_2_O_3_‐based composite systems [22, 23]. Furthermore, chronoamperometric measurements under 40 kHz ultrasound (Figure S5c) reveal current transients for the composite electrode, consistent with cavitation‐driven charge transfer at the heterointerface, whereas the SiO_2_ control exhibits no discernible response. Adsorption‐energy calculations (Figure S4d) indicate that H_2_O is progressively more strongly bound across the series, with E_ads_ = −0.47 eV on TiO_2_, −0.68 eV on Ti_3_C_2_, and −1.03 eV on the TiO_2_/Ti_3_C_2_ heterointerface, providing a favorable thermodynamic precondition for reactant enrichment. It should be noted that these adsorption energies describe the thermodynamic stability of the adsorbed state relative to the gas‐phase species and are distinct from activation energies (e.g., the kinetic barrier for H_2_O dissociation at the heterointerface), which govern the kinetic accessibility of surface reactions. The two quantities are complementary but not interchangeable: strong adsorption facilitates reactant concentration at the interface, whereas the activation barrier determines the rate of subsequent bond‐breaking and bond‐forming steps. The EPR signal at *g* = 2.003 (Figure 1d) captures the signature of paramagnetic defect states in the composite [18]. Taken together with the DFT and electrochemical evidence, these results are consistent with the proposed principle of synergistic interfacial charge separation and defect modulation.

The H_2_O dissociation barrier on pristine TiO_2_(101), obtained by the climbing‐image nudged elastic band (CI−NEB) method, is 1.28 eV (Figure S4e), serving as an upper‐bound reference for the kinetic bottleneck of water activation on unengineered TiO_2_. These DFT results describe the ground‐state thermodynamic predisposition of the heterointerface. However, the actual sonocatalytic environment involves extreme localized conditions during cavitation collapse, including transient high temperatures and pressures and sonoluminescence‐associated excited states [14], that are not captured by ground‐state DFT and may further modulate the reaction pathway under ultrasonic excitation.

Time‐resolved passive cavitation imaging (PCI; Figure 2a and Figure S6 and Texts S1 and S3) was used to compare the spatial distribution of cavitation intensity across four media: pure water, the MAX‐phase precursor (Ti_3_AlC_2_), commercial TiO_2_ (P25), and the MXene/TiO_2_ composite. This set of experiments bridges the electronic‐structure evidence above to the spatial fate of acoustic energy, testing whether the engineered heterointerface translates its charge‐separation advantage into spatially confined cavitation. The MAX and P25 materials were included as negative controls sharing the particulate nature of the composite but lacking its engineered Schottky heterointerface and pre‐existing oxygen‐vacancy/Ti^3^^+^ sites. To conserve space, the full four‐media PCI comparison is presented in the (Figure S6 and Text S3). As quantified therein, the cavitation intensity followed the order MXene/TiO_2_ ≫ P25 ≈ MAX > water, with the composite showing the most persistent interfacial cavitation. The fact that P25 and MAX, both particulate cavitation nuclei, produce cavitation intensities only marginally above water indicates that particle seeding alone contributes only a moderate baseline enhancement. The substantially stronger signal from the composite therefore points to a mechanistic effect specific to the engineered heterointerface. This interpretation is further supported by the chronoamperometric measurements (Figure S5c), in which sharp transient current spikes characteristic of near‐surface cavitation events [11] were observed only for the MXene/TiO_2_ electrode, whereas TiO_2_ exhibited a step‐like on–off current response and the SiO_2_ control showed no discernible response. The concordance between the spatial PCI evidence (consistent with interface‐confined cavitation) and the temporal electrochemical evidence (cavitation‐coupled current transients) suggests that the heterointerface may shape both where and how acoustic energy is converted. Together, the PCI, aluminum‐foil erosion (Figure 2a), and electrochemical data indicate that cavitation coupling to interfacial charge dynamics is most pronounced for the MXene/TiO_2_ heterointerface. Here, aluminum‐foil erosion serves explicitly as a macroscopic cavitation‐energy benchmark. Complementing these experimental observations, computational fluid dynamics (CFD) simulations illustrate the plausible acoustic‐stress distribution and flow‐velocity fields at the solid–liquid interface (Figure 2b and see Text S2 for modeling details and limitations). Within current methodological constraints, the convergence of PCI, electrochemical transients, and quasi‐in situ spectroscopy supplies interface‐resolved corroboration.

**FIGURE 2:**
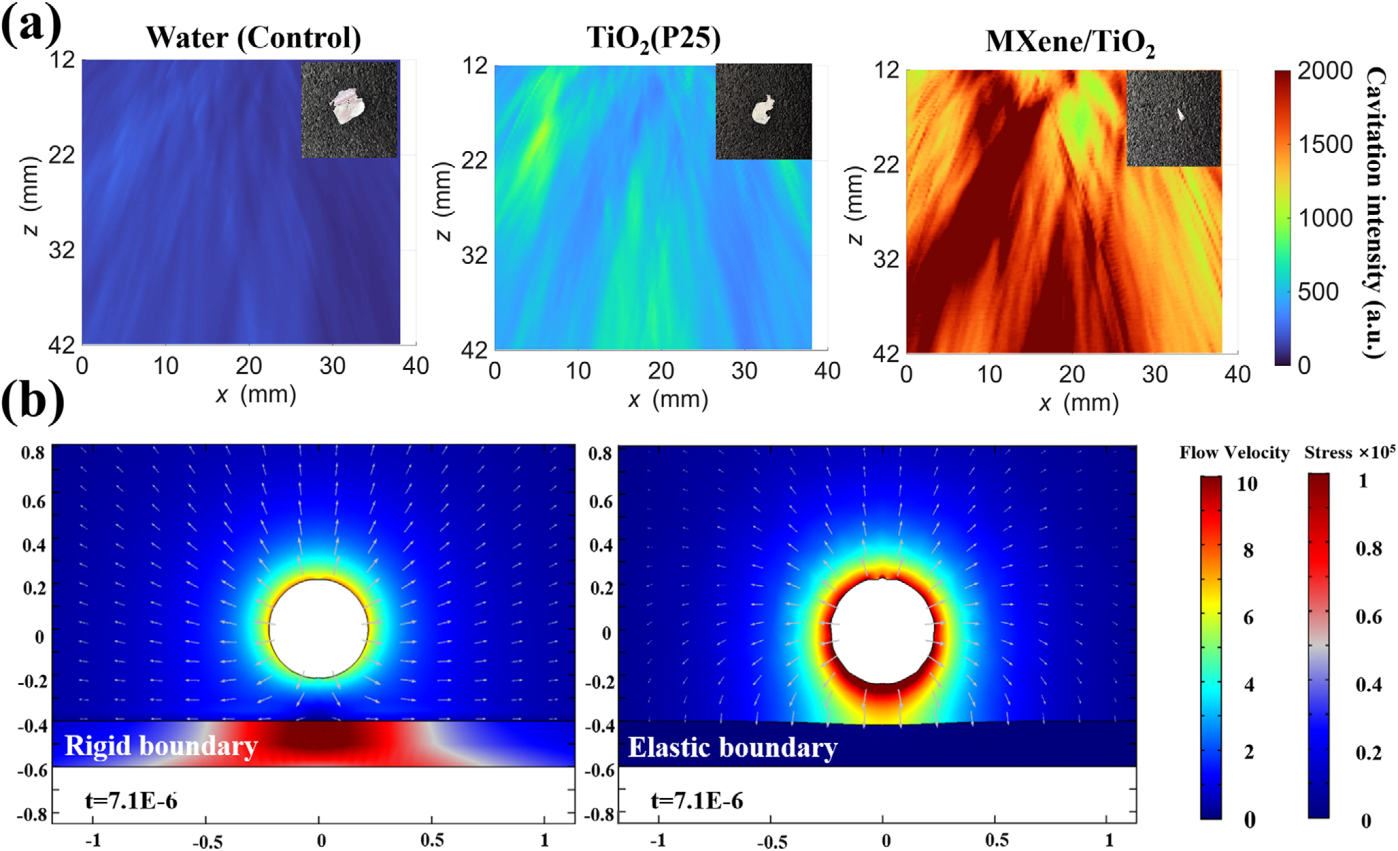
Acoustic cavitation characterization. (a) Passive cavitation imaging (PCI) maps of cavitation‐intensity distributions in water, P25, and MXene/TiO_2_ suspensions under identical ultrasonic irradiation. The insets show representative top‐view photographs of the corresponding eroded aluminum foils. (b) Computational fluid dynamics (CFD) simulation of the flow‐velocity and acoustic‐stress fields at the solid–liquid interface, contrasting a rigid boundary (left) with an elastic boundary representing bacterial cells (right). Passive cavitation imaging (PCI) mapping across all four media (water, MAX, P25, and MXene/TiO_2_) is provided in Figure S6.

To bypass this cell‐envelope‐limited mechanical bottleneck [24, 25], within the working model of interface‐confined cavitation, the following energy‐routing sequence is proposed. We emphasize that the steps described below are mechanistic interpretations supported by convergent indirect evidence (chronoamperometry, quasi‐in situ XPS, and simulations) rather than directly observed processes. The MXene/TiO_2_ Schottky interface is proposed to provide an alternate energy‐routing pathway, in which acoustic energy is hypothesized to be converted into chemical work at the catalyst surface. Rather than depending on random mechanical disruption in the bulk liquid, the hierarchically rough, hydrophilic heterointerface, which is modeled in Figure 2b and corroborated by water contact‐angle characterization in Figure S3b, is proposed to focus cavitation energy at the solid‐liquid boundary. This focusing is illustrated by the single‐bubble CFD model described in Text S2. The peptidoglycan‐rich bacterial envelope acts as a viscoelastic protective barrier against mechanical damage, whereas the rigid, hierarchically structured catalyst surface is hypothesized to concentrate acoustic energy into a confined reaction volume and to enable surface‐confined ROS generation, as an interpretation consistent with the PCI and chronoamperometric evidence. The Schottky built‐in field is proposed to separate the resulting charge carriers before recombination, in line with the reduced charge‐transfer resistance observed by EIS. Oxidative attack on the pathogen is thus interpreted to proceed preferentially at the interface rather than in the bulk; this spatial assignment remains an indirect inference, as direct spatially resolved ROS measurements under sonication were not performed. This proposed redirection from bulk mechanical lysis to interfacial chemical oxidation is consistent with 3‐log pathogen reduction in real juice matrices at the laboratory scale under purely ultrasonic driving at 40 kHz with no extrinsic heating.

In contrast, prior rGO/TiO_2_ photocatalytic decontamination of coconut juice achieved only approximately 0.4 to 0.7 log reduction. More broadly, as summarized in Table S1, most reported ultrasound‐based juice sterilization methods rely on combined treatments such as mild heating, high‐pressure processing, or chemical additives to achieve satisfactory microbial inactivation, often at the expense of heat‐sensitive nutrients. The present 40 kHz/MXene/TiO_2_ system distinguishes itself by attaining 3‐log reduction of both *E. coli* and *S. aureus* in blueberry and celery juices without any thermal co‐processing (Figure S11 and Table S3), while preserving 82% of vitamin C and 88% of total anthocyanin content. A concentration‐dependent analysis further identified 475 mg L^−^^1^ as the most effective tested MXene/TiO_2_ dosage (Figure S12). This comparison underscores the laboratory‐scale antimicrobial efficacy of our sonocatalytic approach in achieving 3‐log inactivation of the tested indicator organisms while maintaining nutritional quality (82% vitamin C and 88% total anthocyanin content in blueberry juice), suggesting potential as a non‐thermal processing candidate for juice preservation, pending validation of food safety, catalyst recovery, and process scale‐up.

These results demonstrate antimicrobial efficacy under laboratory conditions and do not by themselves establish comprehensive food safety or regulatory compliance, which would require additional validation including a broader spectrum of target microorganisms, spoilage and challenge testing, Ti‐speciation and long‐term toxicological studies, and catalyst‐recovery strategies.

The characteristic 1:2:2:1 quartet of the 5,5‐dimethyl‐1‐pyrroline N‐oxide (DMPO)‐·OH adduct [26] and the 1:1:1 triplet of 2,2,6,6‐tetramethylpiperidine (TEMP)‐^1^O_2_ adduct [27] both appear more intense for the MXene/TiO_2_ composite than for the MXene‐only control (Figure S7c,d). This suggests that the heterointerface, rather than MXene alone, is the primary sonocatalytic entity under the tested conditions. Independent time‐dependent UV–vis probe measurements further corroborate these observations (Figure S8). Under 40 kHz/MXene/TiO_2_ treatment, the consumption of 1,3‐diphenylisobenzofuran (DPBF) over 0–10 min indicates singlet‐oxygen‐mediated oxidation, while the progressive oxidative degradation of methylene blue (MB) over 0–20 min is consistent with the generation of additional reactive oxygen species. The composite thereby generates radical species via sonochemical pathways [5], motivating the following working hypothesis: H_2_O dissociatively adsorbs at pre‐existing oxygen vacancies (V_O_), generating surface hydroxyls and potentially reducing adjacent Ti^4^^+^ to Ti^3^^+^.

In this work, quasi‐in situ XPS under an H_2_O atmosphere (Figure 3a,b) provides supportive evidence consistent with this redox transition. In the Ti 2p region (Figure 3a), the Ti^3^^+^ component at ∼458.5 eV increases after 30 min of H_2_O exposure, accompanied by a corresponding decrease in the Ti^4^^+^ (∼459.2 eV) peak. Concurrently, the O 1s spectrum (Figure 3b) shows an enhanced hydroxyl oxygen contribution (∼531.8 eV) relative to lattice oxygen O^2^^−^ (∼530.3 eV). These spectral changes are consistent with water dissociative adsorption at oxygen‐vacancy sites and the concomitant reduction of adjacent Ti^4^^+^ to Ti^3^^+^. Time‐resolved spectral sequences during H_2_O exposure under varied cathodic biases are provided in Figure S9. The concurrent +13% increase in the Ti^3^^+^ component (Figure 3a) and +11% increase in hydroxyl oxygen (Figure 3b) is self‐consistent with water dissociative adsorption at oxygen‐vacancy sites, wherein the generated electron reduces adjacent Ti^4^^+^ to Ti^3^^+^ while the hydroxyl moiety binds to the vacancy. However, because these measurements were performed ex situ under controlled atmosphere and electrical bias as a surrogate for sonication, they support but do not independently demonstrate the proposed defect‐state transition under real ultrasonic operation. These spectral changes represent hypothesized transitions postulated to occur during sonication.

**FIGURE 3:**
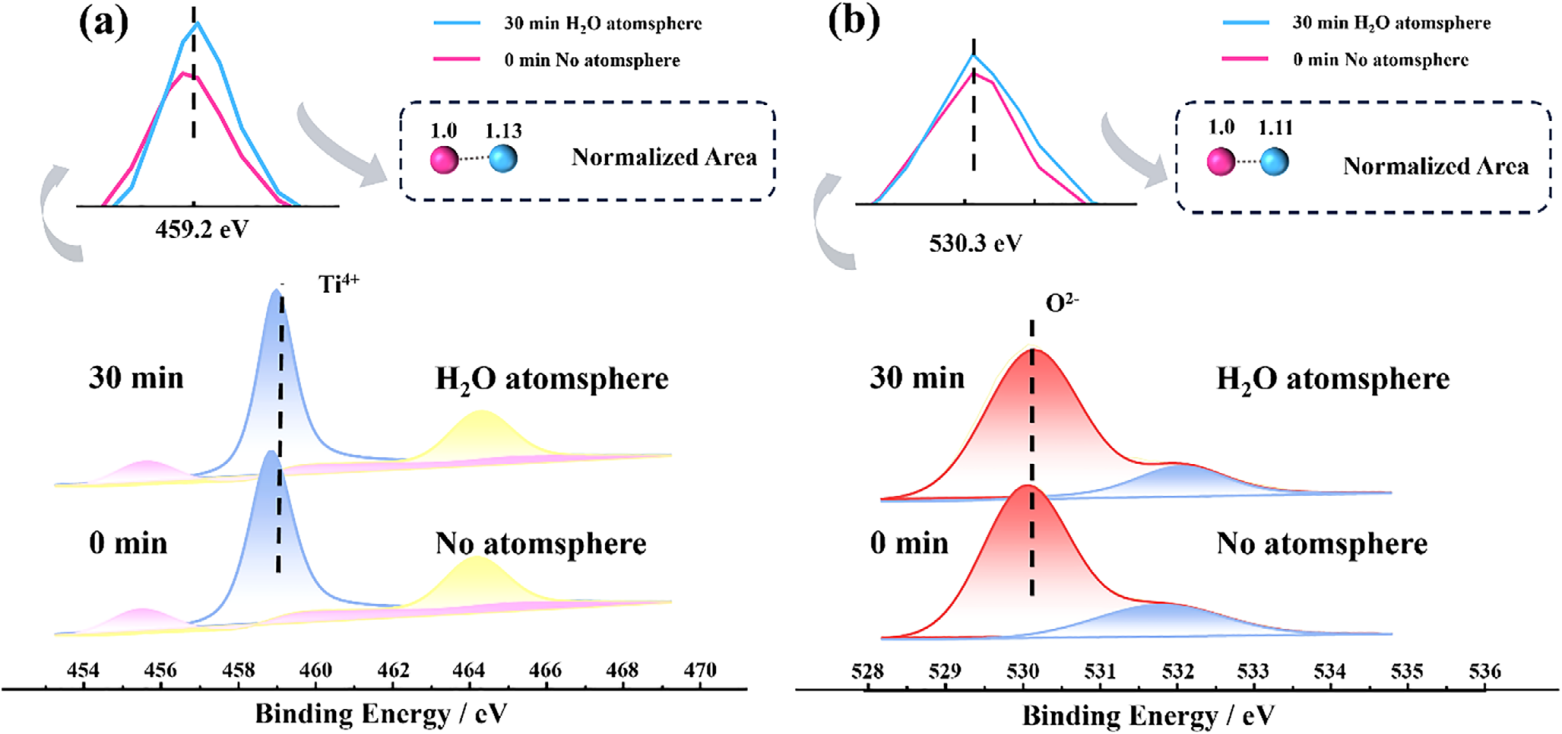
Quasi‐in situ XPS analysis of the evolution of Ti valence states and lattice oxygen on the MXene/TiO2 surface under different atmospheres. (a) Ti 2p and (b) O 1s XPS spectra with corresponding peak deconvolution results, showing the chemical states of the material at the initial state (0 min) and after 30 min of treatment under H2O atmosphere. Enlarged spectral regions highlight the characteristic peaks for lattice oxygen (O^2^^−^, ∼530.3 eV) and Ti^4^^+^ (∼459.2 eV). The Ti^3^^+^ component was constrained to a fixed full‐width at half‐maximum matching the Ti^4^^+^ peak and a spin‐orbit splitting of 5.7 eV, consistent with reported Ti 2p deconvolution protocols. Note: These spectral changes were induced ex situ under controlled atmospheres and bias, representing hypothesized transitions postulated to occur during sonication.

The AIMD trajectories further suggest a plausible sequence of water dissociation followed by proton (H^+^) transfer to an adjacent bridging lattice oxygen, consistent with the increased surface Ti^3^^+^ and hydroxyl signals observed in the quasi‐in situ XPS (Figure 3a,b). This process is visualized through sequential structural snapshots (Figure 4a) and is quantitatively tracked by monitoring the elongation of a critical O‐H bond over time (Figure 4c). The coexistence of both stable molecular water and dissociating species is reflected in the bimodal distribution of O‐H bond lengths sampled during the simulation (Figure 4b).

**FIGURE 4:**
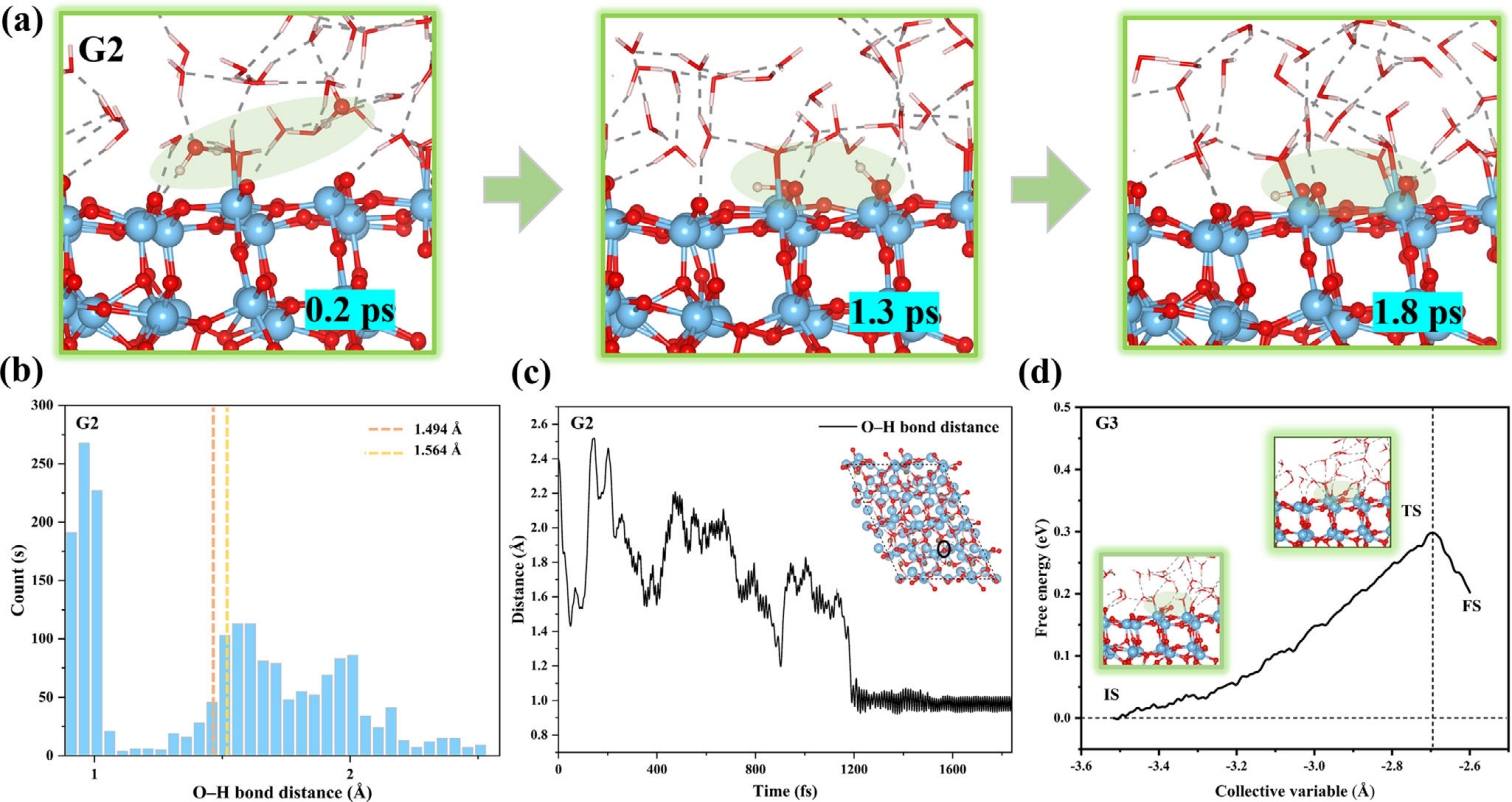
AIMD simulations of water dissociation and proton transfer at the MXene/TiO_2_ heterointerface. (a) Sequential snapshots (0.2, 1.3, and 1.8 ps) illustrating O─H bond elongation and cleavage, with proton transfer to an adjacent bridging oxygen forming terminal and bridging hydroxyls. (b) Bimodal O─H bond length distribution, revealing two distinct states: bound molecular water (short bond) and dissociated interfacial hydroxyls (long separation). (c) Time‐resolved trajectory of a single O‐H bond, showing monotonic elongation culminating in rupture at ∼1.8 ps. (d) Gibbs free‐energy profile of the initial proton‐transfer step, revealing an activation barrier (ΔG) of ∼0.3 eV. Note: the AIMD free‐energy profile (finite temperature, solvated, defect‐engineered interface) is not directly comparable to the static CI‐NEB barrier in Figure S4e (vacuum, defect‐free surface).

To quantify the energetics, the Gibbs free‐energy profile for the initial proton‐transfer step (H_2_O → H^+^ transferred to a bridging lattice O, yielding adsorbed OH^−^) was computed (Figure 4d). The profile reveals an activation barrier of approximately 0.3 eV. Three independent AIMD replicas yielded comparable proton‐transfer free‐energy barriers, supporting the reproducibility of this value (Figure S10). Although this value is significantly higher than the thermal energy at room temperature (k_T_ ≈ 0.025 eV), it may become accessible under the extreme localized conditions generated by acoustic cavitation [28], which are expected to provide sufficient energy input to overcome this barrier.

The ∼0.3 eV barrier corresponds to the initial proton‐transfer step at the simulated, solvated, defect‐engineered interface at 298 K and should not be interpreted as a measured quantity under real sonication. By contrast, the static CI‐NEB barrier of 1.28 eV corresponds to water dissociation on a pristine, defect‐free TiO_2_ (101) surface in vacuum at 0 K. These two values describe different reaction steps under distinct computational conditions and should therefore not be directly compared quantitatively. Nevertheless, the ∼0.3 eV barrier supports the role of the defect‐engineered heterointerface in facilitating the initial proton‐transfer step under the simulated conditions. The calculated ∼0.3 eV value is comparable to previously reported activation barriers for water dissociation at oxide/water interfaces [29, 30, 31], supporting the feasibility of the proposed initial proton‐transfer pathway.

Scavenger‐based quenching experiments allowed an operational partitioning of the relative contributions of the overall bactericidal activity of the 40 kHz/MXene/TiO_2_ system into four operationally defined components: ·OH‐associated, ^1^O_2_‐associated, h^+^‐associated, and residual non‐scavenged contributions. Values are reported as mean ± standard deviation (SD), with n = 3 independent biological replicates per condition. Furfuryl alcohol (FFA, 1 mm), tert‐butanol (1 mm), and ethylenediaminetetraacetic acid (EDTA, 1 mm) were used as scavengers for ^1^O_2_, ·OH, and h^+^, respectively, as described in the Supporting Information [32, 33]. The four components are presented separately in Figure 5. All contributions were normalized to the corresponding scavenger‐free log reduction of the 40 kHz/MXene/TiO_2_ treatment. The residual non‐scavenged contribution was calculated by subtraction and represents other inactivation factors not resolved by the selected scavengers, as detailed in the Supporting Information. For comparison, the corresponding contribution profiles for the ultrasound‐only system are provided in Figure S7a,b. In complex juice matrices, the scavengers may also interact with endogenous antioxidants and other matrix components, potentially affecting the inferred contribution shares. Therefore, the reported percentages are interpreted as relative trends rather than absolute values. In pure water, the combined scavenger‐associated contributions exceeded the residual non‐scavenged contribution. Components such as proteins, lipids, endogenous antioxidants, and pH variations may alter ROS availability and interfacial reactions. Although 3‐log inactivation was achieved in blueberry and celery juices under the tested laboratory‐scale conditions, the effects of individual matrix components and the maintenance of this efficacy at larger processing scales were not independently evaluated. Within the tested blueberry and celery juice systems, the operational contribution profiles remained broadly comparable to those in pure water, with the combined scavenger‐associated contributions exceeding the residual non‐scavenged contribution.

**FIGURE 5:**
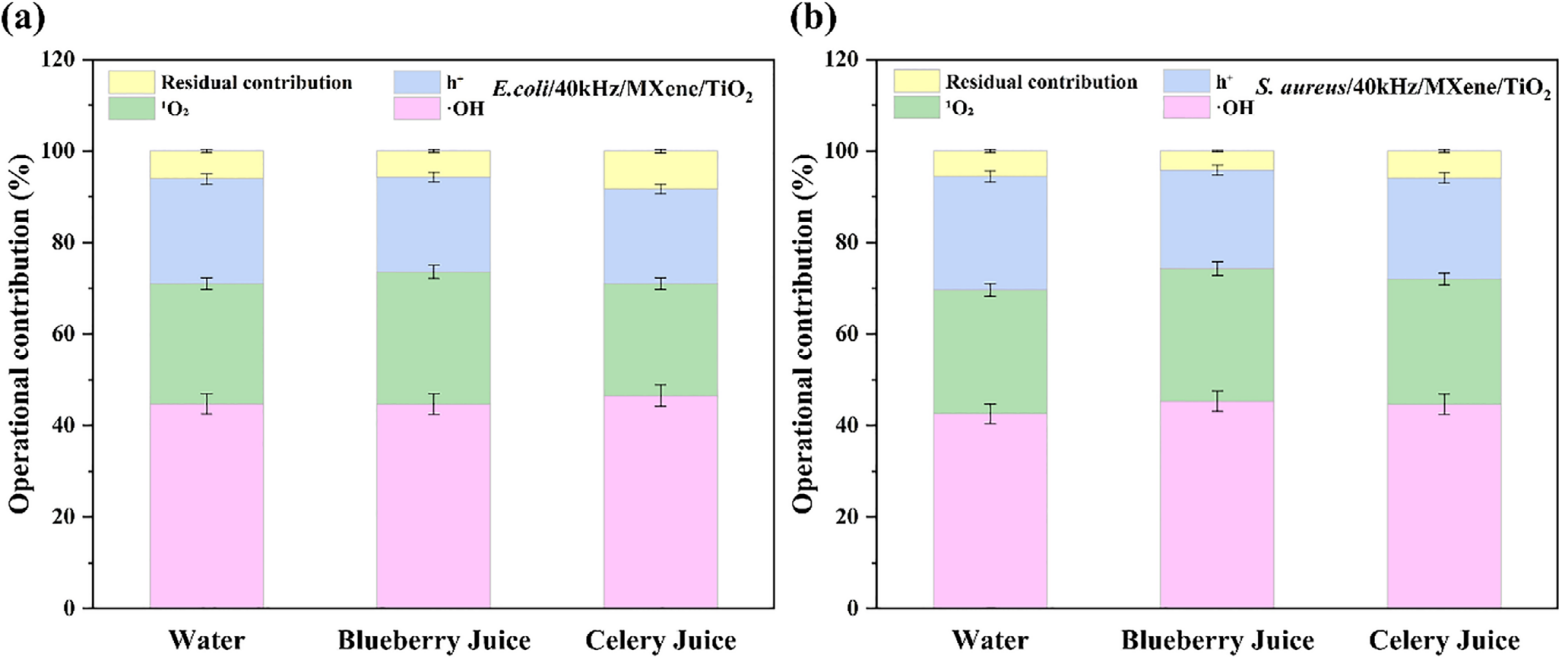
Operational partitioning of relative contributions to bacterial inactivation in different media. (a) E. coli and (b) S. aureus in deionized water, blueberry juice, and celery juice under 40 kHz/MXene/TiO2 treatment. Stacked columns show four operationally defined components: ·OH‐associated, ^1^O2‐associated, h^+^‐associated, and residual non‐scavenged contributions. Furfuryl alcohol (FFA, 1 mm), tert‐butanol (1 mm), and ethylenediaminetetraacetic acid (EDTA, 1 mm) were used as scavengers for ^1^O2, ·OH, and h^+^, respectively. All contributions were normalized to the corresponding scavenger‐free log reduction of the 40 kHz/MXene/TiO2 treatment. The residual non‐scavenged contribution was calculated by subtraction and represents the operational remainder not resolved by the selected scavengers. Calculation details are provided in the Supporting Information, section ‘Reactive‐Species Scavenging Experiments’. The percentages represent operational estimates rather than independently measured mechanistic contributions. Error bars represent SD (n = 3).

The slight negative zeta potential (≈ −2 mV) of the catalyst is consistent with colloidal stability, favoring electrostatic repulsion between MXene/TiO_2_ nanosheets and preventing severe aggregation. Since bacterial membranes (E. coli ≈ −44 mV, S. aureus ≈ −36 mV) also carry negative charges, the observed inactivation is unlikely to be dominated by electrostatic attraction. Two non‐exclusive interpretations may explain the observed redistribution of bactericidal contributions in juice matrices: (i) enhanced local ROS generation at the catalyst‐liquid interface driven by acoustic cavitation, or (ii) changes in scavenger‐associated and residual non‐scavenged contributions under food‐matrix conditions. The scavenger data cannot distinguish between these possibilities or identify the individual factors underlying the residual non‐scavenged contribution.

Consistent with the ROS‐mediated pathway inferred in the juices, flow‐cytometry analysis (Figure S13) provides complementary evidence of bacterial membrane damage. Staining with SYTO 9 reveals a pronounced left‐shift for the 40 kHz/MXene/TiO_2_ group, indicating widespread membrane damage [34]. SEM/TEM morphological characterization further visualizes the associated bacterial cell damage (Figure S14). This single‐cell‐level evidence complements the scavenger‐based bulk deconvolution and is consistent with the interface‐localized, ROS‐mediated inactivation pathway. The surface‐confined radical generation may contribute to the observed shift toward ROS‐dominated killing while preserving heat‐labile nutrients in the bulk phase [2].

In blueberry juice, the treatment preserved 82% of vitamin C and 88% of total anthocyanin content (TAC) (Figure S15a–c), outperforming thermal methods, where vitamin C degradation commonly exceeds 30% [2]. This high retention primarily reflects the non‐thermal process and may also be favored by interface‐localized ROS generation, although its contribution was not isolated here [35]. Chemical fingerprint principal component analysis (PCA) showed treated samples clustering tightly with fresh juice (Figure 6a). Complementary electronic‐nose response patterns and PCA results for blueberry and celery juices are provided in Figure S15d–f and Table S4. Electronic‐tongue analysis indicated that the overall taste profile of blueberry juice was largely retained after 40 kHz/MXene/TiO_2_ treatment, with five of the eight measured attributes remaining statistically comparable to the control, despite selective changes in bitterness, astringency, and aftertaste‐B (Figure 6b and Table S5). The corresponding taste profile of celery juice is provided in Figure S15g. After filtration and microwave digestion, the total Ti concentrations in treated water, blueberry juice, and celery juice were 0.457 ± 0.022, 0.278 ± 0.006, and 0.221 ± 0.002 mg L^−^^1^, respectively, compared with untreated‐matrix background levels of 0.151, 0.056, and 0.081 mg L^−^^1^, respectively (Table S2). Because the digestion‐based inductively coupled plasma mass spectrometry (ICP–MS) method cannot distinguish dissolved Ti from fine Ti‐containing particles, these values represent the total Ti in the filtered samples and provide a preliminary assessment of Ti transfer rather than evidence of food‐safety compliance; no regulatory threshold is currently applicable to catalyst‐derived Ti in treated food products, and no claim of food‐safety compliance can be made from these data alone [36, 37]. As an initial biological evaluation, *Caenorhabditis elegans* exposed for 24 h to the treated juice samples (2%, v/v) exhibited >96% survival and intact morphology comparable to the untreated controls (Figure S16b,c), providing a preliminary safety‐related observation under the tested conditions [38]. These observations do not constitute toxicological validation, and food‐safety compliance has not been established. Further Ti‐speciation studies and long‐term toxicological evaluation remain necessary before practical application; immobilization of MXene/TiO_2_ on a recoverable membrane may offer a strategy to minimize catalyst release and facilitate post‐treatment separation. A preliminary life‐cycle comparison indicated that the sonocatalytic process could reduce production costs and global warming potential relative to high‐pressure processing (Figure S17) [39]. Within the limitations of a laboratory‐scale screening assessment, this positions the process as a promising, energy‐efficient candidate for further development. Over five consecutive 30‐min processing cycles in blueberry juice, bactericidal efficacy showed no statistically significant decline (analysis of variance (ANOVA), p > 0.05; Figure S18). This suggests promising operational stability of the composite under repeated use.

**FIGURE 6:**
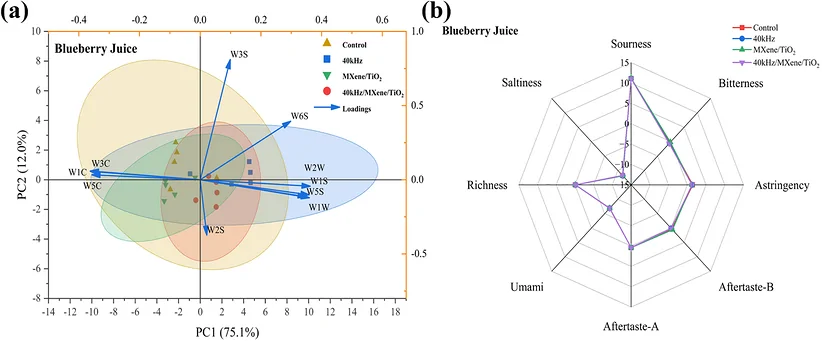
Impact of sonodynamic treatment on bioactive compounds and sensory profiles in fruit and vegetable juices. (a) Principal component analysis (PCA) of blueberry juice samples under different treatments. Score plots illustrate treatment‐dependent variation in the overall chemical fingerprint, with the variance explained by PC1 and PC2 indicated. Each point in panel (a) represents one replicate measurement (n = 5 per treatment). (b) Sensory attribute analysis of blueberry juice under different treatments. The radar plot depicts the multidimensional sensory profile, with each axis representing a specific attribute. Values are plotted as mean ± SD (n = 3 independent juice preparations); error bars are included at each vertex but may be visually obscured in the radar‐plot rendering.

The prevailing view treats the sonocatalyst as a passive source of nucleation sites [13]. This work provides evidence that may challenge that picture. Convergent cavitation‐imaging, electrochemical, and spectroscopic evidence is consistent with the interpretation that the MXene/TiO_2_ heterointerface modulates the spatial distribution of cavitation and the subsequent chemical fate of the deposited energy [13, 14, 40, 41, 42]. This regime is proposed as interface‐confined cavitation: a triad of a Schottky built‐in field for charge separation, a hydrophilic hierarchically rough architecture for energy focusing, and pre‐existing oxygen‐vacancy/Ti^3^^+^ sites for dynamic defect modulation [43]. This triad constitutes the structural principle of the working model. It is proposed to transform the catalyst from a nucleation seed into an energy‐routing platform, and, if confirmed by operando validation, would parallel how heterojunction photocatalysis reframed charge‐separation thinking in the 2010s. The passive‐cavitation‐imaging ranking (MXene/TiO_2_ ≫ P25 ≈ MAX > water) and the aluminum‐foil erosion test provide experimental support consistent with this proposed regime, with the P25 control guarding against a generic particle‐seeding interpretation.

A second contribution reframes the static oxygen‐vacancy narrative. Although the prevailing view treats oxygen vacancies as a static asset whose benefit saturates beyond a threshold concentration [10], quasi‐in situ XPS and AIMD results are consistent with reversibly interconvertible Ti^4+^/Ti^3+^ redox states gated by a proton‐transfer barrier of approximately 0.3 eV. Such defect‐mediated redox behavior, which facilitates charge transfer across the Schottky heterointerface, is consistent with observations in other reducible metal‐oxide systems, including NiO‐, CeO_2_‐, and Fe_2_O_3_‐based materials, where interface/electronic‐structure engineering, mixed‐valence redox couples, and oxygen‐vacancy‐related defects have been associated with improved charge transfer and carrier separation [44, 45, 46]. Five‐cycle stability argues against irreversible vacancy depletion. Consistently, post‐treatment XRD patterns showed that the principal diffraction features of the MXene/TiO_2_ composite were retained after 40 kHz ultrasonication for 30 and 60 min, without the appearance of detectable new crystalline phases (Figure S19). These results further support the phase‐level structural stability of the composite under the tested ultrasonic conditions. The dual‐state redox shuttle remains a working hypothesis, not an operando‐confirmed mechanism, and operando EPR or quasi‐operando XPS will be required to validate it. If confirmed, it resonates with metal‐deficient TiO_2_ and single‐atom Ru/TiO_2__−__x_ sonosensitizers [47, 48, 49, 50, 51, 52, 53, 54, 55]. The third contribution provides evidence on how food matrices reshape cavitation–catalysis coupling. Under identical 40 kHz conditions, the relative partitioning of bactericidal contributions shifts only slightly in antioxidant‐rich juices, and the juice matrix exerts no significant adverse effect on the overall bactericidal activity. This behavior is consistent with interface‐localized chemistry. Bulk radicals are expected to be strongly damped by juice antioxidants [56, 57, 58]. Concomitantly, one plausible explanation is that ROS‐mediated attack occurs predominantly in the vicinity of the catalyst–liquid interface [59]; however, direct spatially resolved measurement of ROS distribution under sonication was not performed: radical generation, bacterial encounter, and oxidative attack are expected to be colocalized, whereas bulk nutrients would remain spatially decoupled from the reactive volume. The observed preservation of 82% vitamin C and 88% total anthocyanin content in blueberry juice is primarily attributable to the non‐thermal nature of the process, and may additionally be favored by an interface‐localized ROS chemistry; the present experiments do not isolate the individual contributions, positioning the system as an energy‐efficient hurdle component within multi‐barrier preservation strategies rather than a stand‐alone terminal sterilizer.

Interface‐confined cavitation is proposed to supply the structural principle, dynamic defect modulation the electronic mechanism, and food‐matrix performance the functional validation. These three layers are deliberately separable, each testable in isolation through targeted Schottky variants, operando spectroscopy, and pilot‐scale food validation. By combining probes that operate under active sonication with surrogate evidence, the multi‐proxy strategy lifts this study beyond an application report toward a design reference for sonocatalytic interfaces. Advancing operando observation of the active interface remains the decisive field‐level step.

## 3 Conclusion

In summary, this work presents an MXene/TiO_2_ Schottky heterointerface as an efficient, non‐thermal sonocatalytic platform for laboratory‐scale decontamination of complex food matrices, delivering a 3‐log reduction of the tested indicator bacteria (*E. coli* and *S. aureus*) while retaining 82% of vitamin C and 88% of total anthocyanin content under mild ultrasonic driving at 40 kHz without external heating. This performance is underpinned by three interconnected advances. First, the concept of interface‐confined cavitation is introduced as a working model, in which a Schottky built‐in field, hierarchical surface roughness, and pre‐existing oxygen vacancies are proposed to collectively favor the conversion of acoustic energy into surface‐associated chemistry rather than its dissipation in the bulk. This paradigm would challenge the prevailing view of the catalyst as a passive nucleation seed, and, if validated, would parallel the conceptual shift that heterojunction photocatalysis brought to charge‐separation thinking in the 2010s. This interface‐engineering strategy may also prove generalizable to other sonocatalytic applications that rely on localized acoustic‐energy conversion and interfacial ROS generation, including pollutant degradation and sonodynamic therapy. Second, the static oxygen‐vacancy narrative is reframed through water‐mediated dynamic defect modulation, in which reversibly interconvertible Ti^4^^+^/Ti^3^^+^ redox states are proposed to facilitate charge transfer across the Schottky heterointerface and may enable a kinetically accessible proton‐transfer gateway for water activation; this dual‐state redox shuttle remains a working hypothesis requiring operando confirmation. Third, food‐matrix validation reveals only a slight redistribution of bactericidal contributions without compromising efficacy. ROS may be generated and consumed near the catalyst–liquid interface, potentially limiting their diffusion into the bulk phase and contributing to the preservation of heat‐labile nutrients. Together, these contributions form a transferable design rationale for sonocatalytic interfaces, positioning the system as an energy‐efficient hurdle component within multi‐barrier preservation strategies. Despite the demonstrated operational stability over five cycles, practical scale‐up will require maintaining uniform cavitation fields in continuous‐flow reactors and developing efficient strategies for catalyst separation and recovery. Although the quasi‐in situ XPS results provide supportive evidence for the proposed dynamic modulation of Ti^4^^+^/Ti^3^^+^ states, definitive confirmation of this redox process under real ultrasonic cavitation will require future operando characterization, which remains a decisive next step for advancing the interfacial engineering of sonocatalysis.

## Author Contributions

**Xiaoge Wu** (X.W.) conceived and designed the study, acquired funding, supervised the project, and wrote the manuscript. **Yunxiao Zhu** (Y.Z.) performed the experiments, interpreted the data, and prepared the figures. **Nan Qin** (N.Q.) synthesized the MXene/TiO_2_ sonosensitizer and participated in part of the experiments. **Qi Zhang** (Q.Z.) mapped the bubble distribution and explored the physical fundamentals of cavitation. **Zhaoli Zhang** (Z.Z.) and **Yang Tao** (Y.T.) guided the food‐related experiments; Y.T. additionally revised the manuscript and acquired funding. All authors reviewed and approved the final manuscript.

## Conflicts of Interest

The authors declare no conflicts of interest.

## Supporting information




**Supporting File**: advs78536‐sup‐0001‐SuppMat.docx.

## Data Availability

The raw data supporting the findings of this study are openly available in figshare at https://doi.org/10.6084/m9.figshare.33120113.

## References

[1] World Health Organization , Estimating the Burden of Foodborne Diseases: A Practical Handbook for Countries (World Health Organization, 2021).

[2] M. C. Giannakourou and P. S. Taoukis , “Effect of Alternative Preservation Steps and Storage on Vitamin C Stability in Fruit and Vegetable Products: Critical Review and Kinetic Modelling Approaches,” Foods 10 (2021): 2630, 10.3390/foods10112630. PMC8619176 34828909

[3] F. Chemat , Z. Huma , and M. K. Khan , “Applications of Ultrasound in Food Technology: Processing, Preservation and Extraction,” Ultrasonics Sonochemistry 18, no. 4 (2011): 813–835, 10.1016/j.ultsonch.2010.11.023. 21216174

[4] K. S. Suslick , “Sonochemistry,” Science 247, no. 4949 (1990): 1439–1445, 10.1126/science.247.4949.1439. 17791211

[5] P. Riesz and T. Kondo , “Free Radical Formation Induced by Ultrasound and Its Biological Implications,” Free Radical Biology and Medicine 13, no. 3 (1992): 247–270, 10.1016/0891-5849(92)90021-8. 1324205

[6] X. Wang , X. Zhong , L. Bai , et al., “Ultrafine Titanium Monoxide (TiO_1+X_) Nanorods for Enhanced Sonodynamic Therapy,” Journal of the American Chemical Society 142, no. 14 (2020): 6527–6537, 10.1021/jacs.9b10228. 32191455

[7] J. Low , J. Yu , M. Jaroniec , S. Wageh , and A. A. Al‐Ghamdi , “Heterojunction Photocatalysts,” Advanced Materials 29, no. 20 (2017): 1601694, 10.1002/adma.201601694. 28220969

[8] G. B. Strapasson , A. S. Arjona , J. E. McPeak , et al., “Oxygen Vacancy‐Induced Phase Transformations of Iron‐Doped Titanium Oxide Nanostructures,” ACS Nano 19, no. 34 (2025): 30986–30999, 10.1021/acsnano.5c08093. PMC12410056 40836394

[9] H. Huang , H. Yan , M. Duan , et al., “TiO_2_ Surface Oxygen Vacancy Passivation towards Mitigated Interfacial Lattice Distortion and Efficient Perovskite Solar Cell,” Applied Surface Science 544 (2021): 148583, 10.1016/j.apsusc.2020.148583.

[10] F. Wang , Q. Zhao , H. Li , et al., “Crystal Defect Engineering to Construct Oxygen Vacancies in MXene‐Derived TiO_2_ Nanocomposites for Boosting Photocatalytic Degradation of 2,4,6‐Trichlorophenol,” Chemical Engineering Journal 481 (2024): 148855, 10.1016/j.cej.2024.148855.

[11] S. Lee , K. C. Devarayapalli , B. Kim , Y. Lim , and D. S. Lee , “Fabrication of MXene‐Derived TiO_2_/Ti_3_C_2_ Integrated With a ZnS Heterostructure and Their Synergistic Effect on the Enhanced Photocatalytic Degradation of Tetracycline,” Journal of Materials Science & Technology 198 (2024): 186–199, 10.1016/j.jmst.2024.02.026.

[12] M. Naguib , M. Kurtoglu , V. Presser , et al., “Two‐Dimensional Nanocrystals Produced by Exfoliation of Ti_3_AlC_2_ ,” Advanced Materials 23, no. 37 (2011): 4248–4253, 10.1002/adma.201102306. 21861270

[13] P. Qiu , B. Park , J. Choi , B. Thokchom , A. B. Pandit , and J. Khim , “A Review on Heterogeneous Sonocatalyst for Treatment of Organic Pollutants in Aqueous Phase Based on Catalytic Mechanism,” Ultrasonics Sonochemistry 45 (2018): 29–49, 10.1016/j.ultsonch.2018.03.003. 29705323

[14] M. Ashokkumar and F. Grieser , “A Comparison Between Multibubble Sonoluminescence Intensity and the Temperature Within Cavitation Bubbles,” Journal of the American Chemical Society 127, no. 15 (2005): 5326–5327, 10.1021/ja050804k. 15826163

[15] D. Zhang , E. Cui , E. Jiang , et al., “Molecular Dynamics Investigation on the Influence of Surface Characteristics on Ultrasonic Cavitation,” Physics of Fluids 38, no. 1 (2026): 012019, 10.1063/5.0310741.

[16] S. K. Sheik Moideen Thaha and P. Sathishkumar , “MXene Based Nanoarchitectures for Organic Contaminants Degradation Under Sonophotocatalytic Environment: Eco‐Friendly Synthesis, Catalytic Attributes and Recent Advancements,” RSC Advances 15, no. 34 (2025): 28093–28120, 10.1039/d5ra04096e. PMC12329656 40778104

[17] R. Yan , Q. Wu , G. Lin , et al., “Inhibition of Salmonella typhimurium and Listeria monocytogenes in Coconut Juice by Graphene‐Doped Photocatalyst rGO/TiO_2_ ,” Food Chemistry 463 (2025): 141103, 10.1016/j.foodchem.2024.141103. 39255706

[18] S. Yang , L. E. Halliburton , A. Manivannan , et al., “Photoinduced Electron Paramagnetic Resonance Study of Electron Traps in TiO_2_ Crystals: Oxygen Vacancies and Ti^3+^ Ions,” Applied Physics Letters 94, no. 16 (2009): 162114, 10.1063/1.3124656.

[19] A. Sarycheva and Y. Gogotsi , “Raman Spectroscopy Analysis of the Structure and Surface Chemistry of Ti_3_C_2_T_x_ MXene,” Chemistry of Materials 32, no. 8 (2020): 3480–3488, 10.1021/acs.chemmater.0c00359.

[20] F. Tian , Y. Zhang , J. Zhang , and C. Pan , “Raman Spectroscopy: A New Approach to Measure the Percentage of Anatase TiO_2_ Exposed (001) Facets,” The Journal of Physical Chemistry C 116, no. 13 (2012): 7515–7519, 10.1021/jp301256h.

[21] R. Ma , S. Rao , J. Xie , et al., “In Situ Construction of Vertical‐TiO_2_/MXene Schottky Heterojunction With Enhanced Bacterial Capture for Synergistic Photothermal and Photocatalytic Antibacterial Effects,” Applied Surface Science 710 (2025): 164011, 10.1016/j.apsusc.2025.164011.

[22] S. Devi , Tripta , Suman , et al., “Enhanced Photocatalytic Activity of α‐Fe_2_O_3_/MgO Nanocomposites for Environmental Sustainability,” Ceramics International 50, no. 13 (2024): 24608–24617, 10.1016/j.ceramint.2024.04.195.

[23] S. Devi , Suman , S. Chahal , et al., “Magnetic Fe_2_O_3_/CNT Nanocomposites: Characterization and Photocatalytic Application towards the Degradation of Rose Bengal Dye,” Ceramics International 49, no. 12 (2023): 20071–20079, 10.1016/j.ceramint.2023.03.130.

[24] Ž. Pandur , M. Dular , R. Kostanjšek , and D. Stopar , “Bacterial Cell Wall Material Properties Determine *E. coli* Resistance to Sonolysis,” Ultrasonics Sonochemistry 83 (2022): 105919, 10.1016/j.ultsonch.2022.105919. PMC8789596 35077964

[25] S. Gao , G. D. Lewis , M. Ashokkumar , and Y. Hemar , “Inactivation of Microorganisms by Low‐Frequency High‐Power Ultrasound: 2. A Simple Model for the Inactivation Mechanism,” Ultrasonics Sonochemistry 21, no. 1 (2014): 454–460, 10.1016/j.ultsonch.2013.06.007. 23845410

[26] G. V. Buxton , C. L. Greenstock , W. P. Helman , and A. B. Ross , “Critical Review of Rate Constants for Reactions of Hydrated Electrons, Hydrogen Atoms and Hydroxyl Radicals (OH/O^−^ in Aqueous Solution,” Journal of Physical and Chemical Reference Data 17, no. 2 (1988): 513–886, 10.1063/1.555805.

[27] L. Cai , Q. Yao , X. Du , et al., “Validation of Quenching Effectiveness and Pollutant Degradation Ability of Singlet Oxygen Through Model Reaction System,” Journal of Hazardous Materials 460 (2023): 132488, 10.1016/j.jhazmat.2023.132488. 37696208

[28] K. S. Suslick and D. J. Flannigan , “Inside a Collapsing Bubble: Sonoluminescence and the Conditions during Cavitation,” Annual Review of Physical Chemistry 59, no. 1 (2008): 659–683, 10.1146/annurev.physchem.59.032607.093739. 18393682

[29] Y. Zhang , Z. Xu , G. Li , X. Huang , W. Hao , and Y. Bi , “Direct Observation of Oxygen Vacancy Self‐Healing on TiO_2_ Photocatalysts for Solar Water Splitting,” Angewandte Chemie International Edition 58, no. 40 (2019): 14229–14233, 10.1002/anie.201907954. 31369193

[30] Y. J. Kim , D. Kim , Y. Kim , Y. Jeong , H.‐K. Lee , and J. Y. Park , “Correlation Between Defect and Lattice States on Modulated TiO_2_(110) during Redox Reaction,” The Journal of Physical Chemistry C 129, no. 4 (2025): 2028–2035, 10.1021/acs.jpcc.4c08280.

[31] B. Wen , M. F. Calegari Andrade , L.‐M. Liu , and A. Selloni , “Water Dissociation at the Water–Rutile TiO_2_ (110) Interface From Ab Initio‐Based Deep Neural Network Simulations,” Proceedings of the National Academy of Sciences 120, no. 2 (2023): 2212250120, 10.1073/pnas.2212250120. PMC9926290 36598953

[32] E. Appiani , R. Ossola , D. E. Latch , P. R. Erickson , and K. McNeill , “Aqueous Singlet Oxygen Reaction Kinetics of Furfuryl Alcohol: Effect of Temperature, pH, and Salt Content,” Environmental Science: Processes & Impacts 19 (2017): 507–516, 10.1039/c6em00646a. 28244514

[33] A. Khataee , S. Saadi , B. Vahid , S. W. Joo , and B.‐K. Min , “Sonocatalytic Degradation of Acid Blue 92 Using Sonochemically Prepared Samarium Doped Zinc Oxide Nanostructures,” Ultrasonics Sonochemistry 29 (2016): 27–38, 10.1016/j.ultsonch.2015.07.026. 26584981

[34] F. Ou , C. McGoverin , S. Swift , and F. Vanholsbeeck , “Rapid and Cost‐Effective Evaluation of Bacterial Viability Using Fluorescence Spectroscopy,” Analytical and Bioanalytical Chemistry 411, no. 16 (2019): 3653–3663, 10.1007/s00216-019-01848-5. PMC6571086 31049617

[35] M. Umair , M. Abid , M. Mumraiz , et al., “Emerging Frontiers in Juice Processing: The Role of Ultrasonication and Other Non‐Thermal Technologies in Enhancing Antioxidant Capacity and Quality of Fruit and Vegetable Juices,” Ultrasonics Sonochemistry 122 (2025): 107554, 10.1016/j.ultsonch.2025.107554. PMC12510019 41016255

[36] S. Dominguez , A. Kassouf , J. Théolier , I. S. Al‐Gharibi , and S. B. Godefroy , “Comprehensive Review on Reported Ti and TiO_2_ Concentrations in Food: Towards a Standardised Threshold for Detecting Intentional Addition,” Food Control 190 (2026): 112407, 10.1016/j.foodcont.2026.112407.

[37] M. Younes and G. Aquilina , “Safety Assessment of Titanium Dioxide (E171) as a Food Additive,” EFSA Journal 19 (2021): 6585, 10.2903/j.efsa.2021.6585. PMC8101360 33976718

[38] F. Zheng , C. Chen , and M. Aschner , “Neurotoxicity Evaluation of Nanomaterials Using *C. elegans*: Survival, Locomotion Behaviors, and Oxidative Stress,” Current Protocols 2, no. 7 (2022): 496, 10.1002/cpz1.496. PMC9299521 35849041

[39] G. Mendes‐Oliveira , A. J. Deering , M. F. San Martin‐Gonzalez , and O. H. Campanella , “Microwave pasteurization of apple juice: Modeling the inactivation of Escherichia coli O157:H7 and Salmonella Typhimurium at 80–90°C,” Food Microbiology 87 (2020): 103382, 10.1016/j.fm.2019.103382. 31948623

[40] K. S. Ranjith , S. M. Ghoreishian , R. Umapathi , et al., “WS_2_‐Intercalated Ti_3_C_2_T_x_ MXene/TiO_2_‐Stacked Hybrid Structure as an Excellent Sonophotocatalyst for Tetracycline Degradation and Nitrogen Fixation,” Ultrasonics Sonochemistry 100 (2023): 106623, 10.1016/j.ultsonch.2023.106623. PMC10585321 37832252

[41] E. Maisonhaute , C. Prado , P. White , and R. Compton , “Surface Acoustic Cavitation Understood via Nanosecond Electrochemistry. Part III: Shear Stress in Ultrasonic Cleaning,” Ultrasonics Sonochemistry 9, no. 6 (2002): 297–303, 10.1016/S1350-4177(02)00089-5. 12404794

[42] G. B. Tezel , K. Arole , D. E. Holta , M. Radovic , and M. J. Green , “Interparticle Interactions and Rheological Signatures of Ti_3_C_2_T_z_ MXene Dispersions,” Journal of Colloid and Interface Science 605 (2021): 120–128, 10.1016/j.jcis.2021.07.068. 34311306

[43] E. Maisonhaute , P. C. White , and R. G. Compton , “Surface Acoustic Cavitation Understood via Nanosecond Electrochemistry,” The Journal of Physical Chemistry B 105, no. 48 (2001): 12087–12091, 10.1021/jp012437e.

[44] G. K. Kiran , S. Singh , N. Mahato , et al., “Interface Engineering Modulation Combined With Electronic Structure Modification of Zn‐Doped NiO Heterostructure for Efficient Water‐Splitting Activity,” ACS Applied Energy Materials 7, no. 1 (2024): 214–229, 10.1021/acsaem.3c02519.

[45] Ankita , S. Chahal , S. Singh , et al., “Preparation of CeO_2_@AC and CeO_2_@NF Nanocomposites for Waste Water Treatment,” Diamond and Related Materials 149 (2024): 111643, 10.1016/j.diamond.2024.111643.

[46] Suman , S. Singh , Ankita , et al., “Photocatalytic Activity of α‐Fe_2_O_3_@CeO_2_ and CeO_2_@α‐Fe_2_O_3_ Core‐Shell Nanoparticles for Degradation of Rose Bengal Dye,” Journal of Environmental Chemical Engineering 9, no. 5 (2021): 106266, 10.1016/j.jece.2021.106266.

[47] Y. Zhu , Q. Hao , H. Zhu , et al., “Thermoelectric Nanoheterojunction‐Mediated Multiple Energy Conversion for Enhanced Cancer Therapy,” ACS Nano 18, no. 50 (2024): 34257–34271, 10.1021/acsnano.4c12261. 39630424

[48] Y. Zhao , B. Yuan , L. Yan , et al., “In Situ Synthesis of Ru/TiO_2−X_@TiCN Ternary Heterojunctions for Enhanced Sonodynamic and Nanocatalytic Cancer Therapy,” Advanced Science 11, no. 4 (2024): 2307029, 10.1002/advs.202307029. PMC10811504 38032117

[49] Q. Zhong , Y. Li , J. Liu , J. Li , and G. Zhang , “In Situ Construction of Ti^3+^ Self‐Doped TiO_2_/Ti_3_C_2_ Schottky Heterojunctions for Highly Selective Photo‐Fenton‐Like Degradation of Organic Pollutants: Surface/Interface Effect and Mechanism Insight,” Applied Surface Science 667 (2024): 160376, 10.1016/j.apsusc.2024.160376.

[50] I. Gul , M. Sayed , F. Rehman , et al., “Unlocking the Potential of Multifunctional and Highly Porous Ti_3_C_2_/TiO_2_@Bi_2_O_3_ – based MXene: Synergetic Photocatalytic Activation of Peroxymonosulfate, Hydrogen Evolution and Antimicrobial Activity,” Applied Catalysis B: Environment and Energy 359 (2024): 124493, 10.1016/j.apcatb.2024.124493.

[51] X. Liu , R. Qian , B. Li , Y. Zhang , and Y. Han , “Sono‐Catalytic Tooth Whitening and Oral Health Enhancement With Oxygen Vacancies‐Enriched Mesoporous TiO_2_ Nanospheres: A Nondestructive Approach for Daily Tooth Care,” ACS Biomaterials Science & Engineering 10, no. 10 (2024): 6634–6647, 10.1021/acsbiomaterials.4c01185. 39348292

[52] C. Wang , S. Wu , C. Mao , et al., “Accelerating Sono‐Piezoelectric Charge Transport for Antibacterial Therapy and Bone Regeneration by Metal‐Deficient TiO_2_ Spin‐Polarization Effect,” Advanced Science 12, no. 30 (2025): 03186, 10.1002/advs.202503186. PMC12376564 40432544

[53] Y. Zhu , D. Wang , C. Du , et al., “Ruthenium Single‐Atom Nanozyme Driven Sonosensitizer With Oxygen Vacancies Enhances Electron–Hole Separation Efficacy and Remodels Tumor Microenvironment for Sonodynamic‐Amplified Ferroptosis,” Advanced Science 12, no. 22 (2025): 2416997, 10.1002/advs.202416997. PMC12165091 40279631

[54] J. S. Jeyaprakash , M. Rajamani , P. Mani , C. Yazhini , S. H. Sonawane , and B. Neppolian , “Highly Efficient Ultrasound‐Driven Ti^3+^‐Doped TiO_2_ With Oxygen Vacancies: A Promising Visible‐Light Catalyst for Complete Sonophotocatalytic Degradation of Tetracycline,” Industrial & Engineering Chemistry Research 63, no. 12 (2024): 5135–5147, 10.1021/acs.iecr.3c04429.

[55] W. Zhu , Y. Sun , K. Xu , et al., “Ultrasmall Manganese‐Doped TiO_2–x_ Overcomes the Efficacy–Safety Dilemma of Sonodynamic Therapy via Tumor Microenvironment‐Responsive Reactive Oxygen Species Amplification and Clearance,” ACS Nano 19, no. 44 (2025): 38440–38456, 10.1021/acsnano.5c11335. 41166608

[56] N. Aktı and S. Yildiz , “Exploring Ultrasound‐Induced Free Radical Formation: A Comparative Study in Water and Sour Cherry Juice Using Glutathione and Terephthalic Acid Indicators,” Ultrasonics Sonochemistry 112 (2025): 107193, 10.1016/j.ultsonch.2024.107193. PMC11683305 39657289

[57] A. Rehman , M. Nadeem , M. A. Murtaza , et al., “Investigating the Ultrasonication‐Induced Changes in the Physicochemical, Phytochemical, Microbial, and Sensory Properties of Cantaloupe–Sugarcane Blend Juice,” Food Science & Nutrition 13, no. 7 (2025): 70519, 10.1002/fsn3.70519. PMC12203413 40585490

[58] M. Zhang , C. Zhou , L. Ma , W. Su , J. Jiang , and X. Hu , “Influence of Ultrasound on the Microbiological, Physicochemical Properties, and Sensory Quality of Different Varieties of Pumpkin Juice,” Heliyon 10, no. 6 (2024): e27927, 10.1016/j.heliyon.2024.e27927. PMC10955300 38515695

[59] S. Halder , K. K. Yadav , R. Sarkar , et al., “Alteration of Zeta Potential and Membrane Permeability in Bacteria: A Study With Cationic Agents,” SpringerPlus 4, no. 1 (2015): 672, 10.1186/s40064-015-1476-7. PMC4633473 26558175

