# Supplementary material for: Interface‐Confined Cavitation at an MXene/TiO2 Schottky Heterointerface for Non‐Thermal Juice Preservation
Source: Adv Sci (Weinh). 2026 Oct 28:e78536. Online ahead of print. doi: 10.1002/advs.78536 (PMC13717118; doi:10.1002/advs.78536)
Supplement: Supplementary file 1 [file ADVS-9999-e78536-s001.docx]

Supporting Information

**Interface-Confined Cavitation at an MXene/TiO₂ Schottky Heterointerface for Non-thermal Juice Preservation**

Xiaoge Wu^1‡^, Yunxiao Zhu^1‡^, Nan Qin^1^, Zhaoli Zhang^2*^, Qi Zhang^3*^, Yang Tao^4,5*^

^1^Key Laboratory of Arable Land Quality Monitoring and Evaluation (Yangzhou University), Ministry of Agriculture and Rural Affairs, School of Environmental Science and Engineering, Yangzhou University, Yangzhou 225127, China, ^2^Key Laboratory of Chinese Cuisine Intangible Cultural Heritage Technology Inheritance, College of Tourism and Culinary Science, Yangzhou University, Yangzhou 225127, China, ^3^School of Computer and Electronic Information, Nanjing Normal University, Nanjing 210023, China, ^4^College of Food Science and Technology, Nanjing Agricultural University, Nanjing 210095, Jiangsu, China, ^5^Food Safety and Nutrition Experimental Teaching Demonstration Center, College of Food Science and Engineering, Xinjiang Institute of Technology, Aksu, Xinjiang 843100, China

^‡^Xiaoge Wu and Yunxiao Zhu contributed equally to this work and share first authorship.

**Corresponding authors**

Zhaoli Zhang: zhaoli@yzu.edu.cn

Qi Zhang: qizhang@njnu.edu.cn

Yang Tao: yang.tao@njau.edu.cn

**Materials and Methods**

**Juice Preparation**

Fresh blueberries (*Vaccinium corymbosum* L., cv. Bluecrop, soluble solids 12–14 °Brix) or fresh celery stalks (*Apium graveolens* L., cv. Pascal celery, moisture >90%) were washed with distilled water, blended with deionized (DI) water at 1:4 (m/v), and filtered through four layers of sterile medical gauze. The clarified juice was inoculated with *E. coli* or *S. aureus* to ~10⁷ colony-forming units (CFU) mL⁻¹ and divided into: (i) Control (40 kHz ultrasound alone); (ii) 475 mg L⁻¹ MXene/TiO₂ + 40 kHz ultrasound; (iii) HT100 (100 °C thermal). Juices were stored at 4 °C and used within 2 h.

**Synthesis and Characterization for Functional Evaluation**

All steps involving hydrofluoric acid (HF) were performed in a fume hood with full personal protective equipment. Because addition of Ti₃AlC₂ to HF is strongly exothermic, 2 g of Ti₃AlC₂ MAX (<38 μm, Forsman Scientific, Beijing) was added in small aliquots (~0.2 g) to 20 mL of 40 wt% HF under stirring at room temperature for 24 h to selectively remove the Al layer. The product was washed with ethanol and deionized water by centrifugation (5000 × g) until the supernatant reached pH ~6, then dried at 60 °C for 12 h. Multilayer Ti₃C₂ (200 mg) was dispersed in 30 mL DI water by ultrasonication (100 W, 10 min, ice bath), then heated in a 100 mL Teflon-lined autoclave at 180 °C for 20 h. The product was collected, washed, and vacuum-dried at 60 °C. Scanning electron microscopy (SEM) (Regulus-8100, Hitachi, 5 kV), Transmission electron microscopy/high-resolution TEM (TEM/HRTEM) (Tecnai G2 F30 S-TWIN, FEI, 300 kV), X-ray diffraction (XRD) (D8 ADVANCE, Bruker, Cu Kα λ = 1.5406 Å, 2θ 5–90°), X-ray photoelectron spectroscopy (XPS) (ESCALAB 250Xi, Thermo, Al Kα 1486.6 eV, calibrated to C 1s at 284.8 eV), and Raman (inVia, Renishaw, 532 nm) were used for structural characterization.

**Quasi-In Situ XPS with Cathodic Bias Surrogate**

Since ultrasound cannot be introduced into the XPS vacuum chamber, a cathodic bias (−0.5 to −1.0 V vs. Ag/AgCl) was applied during acquisition to populate Ti³⁺/oxygen-vacancy states, as a surrogate for ultrasound-generated charge carriers. Ti 2p and O 1s spectra were collected under H_2_O vapor (reductive) atmospheres. The sample was pre-reduced (−1.0 V, 5 min, 0.1 M Na₂SO₄) before H₂O exposure to enhance the activation signal. Spectra were acquired at 0 and 30 min. Because the cathodic bias is physically dissimilar to acoustic cavitation, the observed chemical-state evolution supports, but does not independently demonstrate, reversible defect transitions under real sonication; operando validation remains to be performed.

**Density Functional Theory (DFT) Calculations**

DFT was performed with VASP 6.3.2 using the Projector augmented-wave (PAW) method and Perdew–Burke–Ernzerhof (PBE) exchange-correlation, augmented with DFT+U (Dudarev formalism, *U*_eff_ = 3.5 eV on Ti 3d) to reproduce the anatase bandgap (~3.2 eV) and stabilize Ti³⁺/oxygen-vacancy states. DFT-D3 dispersion corrections were applied. The interface was modeled as a Ti₃C₂ (2×2) supercell in contact with an anatase TiO₂ (101) four-layer slab (15 Å vacuum); a plane-wave cutoff of 520 eV and a 3×3×1 Γ-centered k-point mesh were used. Geometry optimization converged to <0.02 eV Å⁻¹. Work functions, PDOS, and d-band centers were computed for pristine TiO₂, Ti₃C₂, and the heterointerface. Adsorption energies were calculated as *E*_ads_ = *E*_total_ − (*E*_slab_ + *E*_adsorbate_): H₂O on TiO₂ (−0.47 eV), on Ti₃C₂ (−0.68 eV), and on the TiO₂/Ti₃C₂ heterointerface (−1.03 eV). GGA-PBE without +U underestimates the bandgap and fails to localize excess electrons at Ti³⁺; all d-band/PDOS conclusions are therefore reported at the DFT+U level. For the mechanism, the H₂O dissociation barrier on a pristine, defect-free TiO₂(101) surface (vacuum, 0 K) was obtained by the climbing-image nudged elastic band (CI-NEB) method as 1.28 eV, serving as the upper-bound reference (Figure S4e). This is the same value referred to in the main text.

**Ab Initio Molecular Dynamics (AIMD)**

AIMD simulations examined the thermodynamic accessibility of the initial proton-transfer step at the MXene/TiO₂ heterointerface. Simulations were performed in the canonical ensemble with a constant number of particles, volume, and temperature (NVT) at 298 K using a Nosé–Hoover thermostat (damping time 1 ps⁻¹) with a time step of 1 fs. The O–H bond evolution of adsorbed water was monitored, and bond-length distributions were sampled at 10 fs intervals. The Gibbs free-energy profile for proton transfer was computed by the blue-moon ensemble method with a collective coordinate defined as the difference between breaking and forming O–H distances. For the bimodal O–H distance distribution shown in Figure 4b, three independent AIMD runs were initiated from different starting configurations of the water/MXene-TiO₂ interface. Each replica was propagated for 1.8 ps, giving a total aggregated trajectory length of 5.4 ps. The bimodal features (~1.0 Å for bound molecular water, ~1.8 Å for dissociated interfacial hydroxyls) and the bond-dissociation event (Figure 4c) were consistently reproduced across all three replicas, indicating that the observed distribution is unlikely to be an artifact of a single initial configuration. Systematic convergence tests (e.g., extending individual trajectories beyond 1.8 ps or performing additional replicas) were not conducted in this study. However, AIMD simulations of water/TiO₂ interfaces in the literature commonly employ per-replica trajectory lengths of 1–5 ps to capture proton-transfer dynamics. The total 5.4 ps sampling therefore falls within the conventionally accepted range for such systems. Statistical representativeness is approximated by independent repetitions and cross-referenced literature precedents; formal convergence testing was not performed. Three independent AIMD runs from different initial configurations yielded activation free energies of 0.298, 0.318, and 0.251 eV, respectively (Figure S10). The mean value of ~0.3 eV is reported in the main text and is consistent with literature proton-transfer barriers at TiO₂/water interfaces [28–31]. For reference, the static CI-NEB barrier of 1.28 eV (Figure S4e) describes H₂O dissociation on a pristine, defect-free TiO₂ surface in vacuum at 0 K, whereas the AIMD profile describes the dynamic, solvent-assisted proton-transfer step at the defect-engineered heterointerface at 298 K. These describe different physical processes and are not directly comparable.

**Bacterial Culture**

Escherichia coli ATCC 25922 and Staphylococcus aureus CMCC(B) 26003 (China Center of Industrial Culture Collection) were grown in Luria–Bertani (LB) broth at 37 °C with shaking (110 rpm, 12 h), harvested by centrifugation, washed three times with sterile 0.9% saline, and adjusted to the desired concentration.

**Sterilization Experiments**

Bacteria (~1.0 × 10⁶ CFU mL⁻¹) were suspended in 20 mL sterile 0.9% saline with 475 mg L⁻¹ MXene/TiO₂ and irradiated with 40 kHz ultrasound in a thermostated bath (WN-020S, 120 W). The working concentration of 475 mg L⁻¹ was selected based on preliminary concentration-dependent antibacterial experiments against *E. coli* under 40 kHz ultrasound (Figure S12). Among the tested concentrations of 250, 375, 425, and 475 mg L⁻¹, 475 mg L⁻¹ exhibited the strongest bactericidal performance and was therefore used in the subsequent experiments. Nonthermal conditions were maintained by external circulating cooling (25 ± 2 °C; sample temperature rise <3 °C over 30 min). Aliquots (100 μL) were collected at 5–10 min intervals, diluted, and spread-plated on LB agar; colonies were counted after 18 h at 37 °C. Bactericidal efficacy was calculated as log reduction = log₁₀(N₀/Nₜ), where N₀ and Nₜ are viable counts of control and treated samples, respectively. Bacteria (~10⁷ CFU mL⁻¹) in 20 mL clarified juice with 475 mg L⁻¹ MXene/TiO₂ were treated as above. Treatment time was 30 min for blueberry juice and 60 min for celery juice (higher organic load/viscosity). Samples were plated as described. Juice or water inoculated with the target bacterium was held at 100 °C for varying durations, cooled on ice, and assessed by plate counting.

**Reactive-Species Scavenging Experiments**

Furfuryl alcohol (FFA, 1 mM), ethylenediaminetetraacetic acid (EDTA, 1 mM), and tert-butanol (1 mM) were used as scavengers for ¹O₂, h⁺, and ·OH, respectively. All scavenging experiments were performed under identical catalyst dosage, ultrasound conditions, treatment time, and bacterial concentration to those of the scavenger-free control. Bactericidal efficacy was expressed as the log reduction, *L*=log_10_ (*N*_0_/*N*_t_). The inhibitory effect associated with each scavenger was calculated as Δ*L*_i_=*L*_0_-*L*_i_, where *L*_0_ is the log reduction without scavenger and *L*_i_ is that measured in the presence of the corresponding scavenger. The relative contribution associated with each scavenger was calculated as *C*_i_ = (Δ*L*_i_/*L*_ref_) × 100%, where *L*_ref_ is the scavenger-free log reduction of the 40 kHz/MXene/TiO₂ treatment for the corresponding bacterial species and medium. The residual non-scavenged contribution was calculated by subtraction as *C*_res_ = [(*L*_0_ − Δ*L*_·OH_ − Δ*L*_¹O₂_ − Δ*L*_h⁺_)/*L*_ref_] × 100%. This residual represents the operational remainder attributed to other inactivation factors not resolved by the selected scavengers. The resulting percentages are operational relative contributions inferred from scavenger-quenching experiments and should not be interpreted as absolute concentrations or yields of the corresponding reactive species. Both the ultrasound-only and 40 kHz/MXene/TiO₂ treatments were normalized to the scavenger-free log reduction of the 40 kHz/MXene/TiO₂ treatment for the corresponding bacterial species and medium. The four components therefore sum to 100% for 40 kHz/MXene/TiO₂, whereas their sum for ultrasound alone equals (*L*_0,US_/*L*_ref_) × 100%. Here, *L*_0,US_ is the scavenger-free log reduction of the ultrasound-only treatment. This operational partition does not establish independent, additive mechanistic contributions or imply that all bactericidal mechanisms were captured by the scavenger assays.

**Electrochemical Measurements**

Electrochemical impedance spectroscopy (EIS) was performed on a CHI 660E workstation (Chenhua) with a three-electrode setup (Ag/AgCl reference, Pt counter, glassy carbon working electrode (GCE), 3 mm). Catalyst ink (4 mg sonosensitizer; 50 μL 5 wt% Nafion; 375 μL DI water; 125 μL ethanol) was drop-cast onto the polished GCE (~0.14 mg cm⁻²) and air-dried.

**Electron Paramagnetic Resonance (EPR) Spectroscopy**

5,5-dimethyl-1-pyrroline N-oxide (DMPO) (50 mM) trapped •OH (1:2:2:1 quartet) [26]; **2,2,6,6-**tetramethylpiperidine (TEMP) (50 mM) detected ¹O₂ (1:1:1 triplet) [27]. Spectra were recorded on a Bruker EMXplus (9.86 GHz, X-band, room temperature). Spin-trap concentrations and an ultrasound-only baseline were included.

**Juice Quality Analysis**

Vitamin C was determined by iodometric titration (expressed as mg ascorbic acid per 100 mL). Total anthocyanin was evaluated by the pH-differential method (malvidin-3-glucoside equivalents). E-nose and e-tongue profiles were recorded for chemical fingerprinting.

**Sensory Evaluation**

A trained panel of nine assessors (Food Science, Yangzhou University) scored 10 attributes covering color, aroma, taste, and overall acceptability on a 0–10 intensity scale, in random-coded presentation with controlled lighting/temperature and water rinses between samples.

**Food Safety Assessment**

Treated samples were microwave-digested, and total Ti was determined by inductively coupled plasma mass spectrometry (ICP–MS) (Agilent 7850). External calibration and reagent blanks were included. Blank-water and ultrasonication-only controls gave 0.151 and 0.293 mg L⁻¹ Ti, indicating a background contribution from non-catalyst sources; reported values (0.221–0.457 mg L⁻¹) are therefore upper-bound estimates of catalyst-derived Ti. The background Ti concentrations in untreated blueberry and celery juices were 0.056 and 0.081 mg L⁻¹, respectively. After 40 kHz/MXene/TiO₂ treatment, the corresponding net increases relative to the untreated juice backgrounds were 0.222 and 0.140 mg L⁻¹, respectively. These measurements provide preliminary safety-related observations, and food-safety compliance has not been established. Synchronized L4-stage *C. elegans* (N2) were exposed for 24 h to 2% (v/v) juice (concentration minimizing effects on survival), with and without MXene/TiO₂, in 1 mL volumes (~30 ± 3 worms/well, three replicates). Survival was scored by absence of movement and response to platinum-wire stimulation.

**Environmental and Cost Assessment**

A laboratory-scale comparative life cycle assessment (LCA) (SimaPro 9.0, ReCiPe 2016 Midpoint (H)) was conducted for sonocatalytic treatment versus high-pressure processing (HPP, 600 MPa, 5 min), using 100 mL juice as the functional unit and global warming potential (GWP) (kg CO₂ eq) as the indicator. A bottom-up screening cost assessment compared direct processing costs under the same functional unit. Because the ultrasonic data are laboratory-scale (120 W bath, 20 mL batches) against literature-reported industrial HPP figures, the resulting GWP and cost values represent laboratory-scale potential indicators rather than industrial feasibility evidence.

Statistical Analysis

Data are expressed as mean ± standard deviation (SD) of three independent replicates. One-way analysis of variance (ANOVA) with Tukey honestly significant difference (HSD) post-hoc testing was applied where appropriate; partial η² values are reported when applicable.

**Table of Contents**

Supplementary Figures

Figure S1. Structural and chemical characterization of MXene and the MXene/TiO₂ composite.

Figure S2. Interface-resolved HRTEM image of the MXene/TiO₂ composite.

Figure S3. N₂ physisorption isotherms and water-contact-angle measurements of the MXene/TiO₂ composite.

Figure S4. Density functional theory (DFT) calculations.

Figure S5. Complementary electrochemical characterizations of the catalysts.

Figure S6. Passive cavitation imaging (PCI) mapping. Quantitative analysis of cavitation intensity at different time points (0 s, 0.72 s, 1.44 s) for water, MAX, P25, and MXene/TiO₂ systems.

Figure S7. Relative antibacterial contribution analysis and direct detection of reactive species.

Figure S8. Time-dependent UV–vis probe measurements of reactive-species generation under 40 kHz/MXene/TiO₂ treatment.

Figure S9. Quasi-in situ XPS measurements of TiO₂−x during exposure to an H₂O atmosphere.

Figure S10. Three independent AIMD-derived proton-transfer free-energy profiles at the MXene/TiO₂ heterointerface.

Figure S11. Antibacterial kinetics.

Figure S12. Concentration-dependent bactericidal kinetics of the MXene/TiO₂ system in water against *E. coli*.

Figure S13. Flow cytometric analysis of bacterial membrane integrity using SYTO 9 staining after different treatments.

Figure S14. Morphological characterization of bacterial cells before and after treatment.

Figure S15. Impact of sonodynamic treatment on bioactive compounds and sensory profiles in fruit and vegetable juices.

Figure S16. Effects of different treatment systems on the survival and morphology of *Caenorhabditis elegans*.

Figure S17. Comparative life-cycle assessment (LCA) of sonocatalytic (40 kHz/MXene/TiO₂) and high-pressure processing (HPP) for juice sterilization.

Figure S18. Reusability of the MXene/TiO₂ sonosensitizer.

Figure S19. XRD patterns of P25, MXene, and MXene/TiO₂ before and after 40 kHz ultrasonic treatment.

**Supplementary Tables**

Table S1. Comparison of reported ultrasound-based juice sterilization methods and the present 40 kHz/MXene/TiO₂ system.

Table S2. Total Ti concentrations in water, blueberry juice, and celery juice following different treatments.

Table S3. Bacterial inactivation at the final treatment time under different treatments.

Table S4. Electronic-nose sensor responses of blueberry and celery juices under different treatments.

Table S5. Electronic-tongue responses of blueberry and celery juices under different treatments.

Supplementary Texts

Text S1. Experimental details for 2D Passive Cavitation Imaging (PCI).

Text S2. Fluid-Solid Coupling Model for Cavitation Bubbles near a Solid Interface (COMSOL Simulation).

Text S3. Description of Time-Resolved Cavitation Imaging and Single-Bubble CFD Analysis.

**Supplementary References**

**S1.0 Figure**

**Figure S1.** Structural and chemical characterization of MXene and the MXene/TiO₂ composite. (a) FE-SEM images at increasing magnifications show the layered, accordion-like morphology of the HF-etched MXene. Yellow dashed boxes outline the regions magnified in the subsequent panels. (b) XRD patterns of the 40% HF-etched MXene and the final MXene/TiO₂ composite, confirming the phase transformation and the presence of anatase TiO₂. (c) Raman spectra displaying the characteristic D and G bands of the carbon structure and the fingerprints of the anatase TiO₂ phase.

**Figure S2.** Interface-resolved HRTEM image of the MXene/TiO₂ composite. The lattice spacings of 0.260 and 0.353 nm are assigned to the MXene-related lattice plane and the anatase TiO₂ (101) plane, respectively. The spatially adjacent lattice fringes demonstrate intimate nanoscale contact between the two components.

**Figure S3**. Porosity and Surface Wettability Characterization of MXene/TiO₂ Composites. (a) N₂ adsorption isotherms and corresponding Brunauer-Emmett-Teller (BET) specific surface areas. The main panel displays the adsorption branches of the isotherms for the MAX phase (Ti₃AlC₂, black squares), MXene (Ti₃C₂, red circles), and the MXene/TiO₂ composite (blue triangles). The composite exhibits the highest N₂ uptake capacity across the entire relative pressure range. The isotherms of all three materials exhibit characteristics of Type I with a steep uptake at low pressure, while the upward trend at high relative pressure (P/P₀ > 0.8) suggests the presence of meso- or macroporosity. The inset bar chart quantifies the BET specific surface areas, confirming that the MXene/TiO₂ composite possesses the largest surface area (8.81 m² g⁻¹), followed by MXene (5.59 m² g⁻¹) and the pristine MAX phase (2.06 m² g⁻¹). The enhanced surface area of the composite correlates with its superior adsorption properties. (b) Water contact angle images comparing the wettability of the substrate and the composite. The upper panel shows a water droplet on the glass substrate with large contact angles (Left: 58.6°, Right: 57.8°), indicating a hydrophobic or weakly hydrophilic nature. In contrast, the lower panel displays the MXene/TiO₂ composite, where the water droplet rapidly infiltrates the surface, demonstrating superhydrophilic behavior. This excellent hydrophilicity facilitates water diffusion into the surface structure, providing qualitative support for the solvated-interface assumption adopted in the AIMD simulations.

**Figure S4**. First-principles investigation of pristine TiO₂, pristine Ti₃C₂, and the TiO₂/Ti₃C₂ heterointerface. (a–c) Optimized structural models, calculated density of states (DOS), and projected density of states (PDOS) of Ti**-**3d orbitals, respectively. The corresponding d-band centers (εd) are −1.52, −0.38, and −0.31 eV for pristine TiO₂, pristine Ti₃C₂, and the TiO₂/Ti₃C₂ heterointerface, respectively. (d) Optimized H₂O adsorption configurations (upper panels) and corresponding charge-density-difference maps (lower panels) for the three systems, with adsorption energies (E_ads_) of −0.47, −0.68, and −1.03 eV, respectively. Yellow and cyan isosurfaces represent electron accumulation and depletion, respectively. (e) Calculated kinetic barrier (activation energy, Eₐ) for H₂O dissociation on the pristine TiO₂(101) surface (Eₐ = 1.28 eV), serving as a benchmark for comparison.

**Figure S5**. **Complementary electrochemical characterizations of the catalysts.** (a) Electrochemical impedance spectra (EIS) Nyquist plots. A smaller arc radius indicates a lower interfacial charge-transfer resistance (Rct) for the MXene/TiO₂ heterojunction compared to pristine MAX and P25. (b) Mott-Schottky (M-S) plots showing the relationship between the space-charge-layer capacitance and the applied potential. The negative shift in the flat-band potential for MXene/TiO₂ is consistent with a reduced Schottky barrier height at the heterointerface compared to pristine TiO₂. (c) Ultrasound-induced current responses are observed for both MXene/TiO₂ and TiO₂, while sharp transient current spikes are observed only for MXene/TiO₂. The SiO₂ control exhibits no discernible response. It should be noted that individual cavitation events occur on microsecond timescales (acoustic period ≈ 25 μs), which cannot be resolved by the millisecond-scale sampling used here. The recorded transients are therefore interpreted as ensemble-averaged indicators rather than direct nanoscale-resolved records. (d) Schematic illustration of the proposed band alignment and interfacial charge-transfer pathways in the MXene/TiO₂ heterojunction under 40 kHz ultrasound.

**Figure S6**. Time-resolved mapping of cavitation intensity distribution in different media. High-resolution color maps depict the spatiotemporal evolution of cavitation activity over three time points (t = 0 s, 0.72 s, 1.44 s) in four media: Water, MAX, P25, and the MXene/TiO₂ composite. Each row corresponds to a specific medium, and each column represents a snapshot in time. The cavitation intensity is represented by a color scale from blue (low) to red (high) in arbitrary units (a.u.). The spatial coordinates (x and z) are in millimeters. The MXene/TiO₂ composite consistently shows the most intense and concentrated cavitation zones (predominantly red/orange) compared to the other materials, particularly at the later time points. This is consistent with more intense and spatially concentrated cavitation associated with the composite structure, in line with its enhanced sonocatalytic performance, and supports the proposed interface-confined cavitation behavior as a mechanistic interpretation. All PCI maps were processed using the same procedure and displayed using an identical intensity scale of 0–2000 a.u.

**Figure S7.** Relative antibacterial contribution analysis and direct detection of reactive species. (a, b) Relative contributions associated with ·OH, ¹O₂, and h⁺, together with the residual non-scavenged contribution, to bacterial inactivation for (a) *E. coli* and (b) *S. aureus* under 40 kHz treatment in water, blueberry juice, and celery juice. All contributions were normalized to the scavenger-free log reduction of the 40 kHz/MXene/TiO₂ treatment for the corresponding bacterial species and medium. The residual non-scavenged contribution was calculated by subtraction and represents the operational remainder not resolved by the selected scavengers. The percentages represent operational estimates rather than independently measured mechanistic contributions or absolute concentrations or yields. (c, d) EPR spectra under 40 kHz irradiation: (c) DMPO–·OH (1:2:2:1 quartet) and (d) TEMP–¹O₂ (three-line signal). The 40 kHz/MXene/TiO₂ system (red) shows markedly higher signal intensities than 40 kHz/MXene (black), indirectly supporting enhanced ·OH and ¹O₂ generation by the heterojunction. Error bars represent SD (n = 3).

**Figure S8.** Time-dependent UV–vis probe measurements of reactive-species generation under 40 kHz/MXene/TiO₂ treatment. (a) Changes in the absorption spectrum of 1,3-diphenylisobenzofuran (DPBF) during 0–10 min of treatment, consistent with the involvement of singlet oxygen and/or other oxidative species. (b) Changes in the absorption spectrum of methylene blue (MB) during 0–20 min of treatment, serving as a non-specific indicator of overall oxidative activity. The decreasing characteristic absorbance of both probes is consistent with enhanced reactive-species generation during sonocatalytic treatment. Control experiments without ultrasound or catalyst showed negligible probe loss over the same periods.

**Figure S9**. Quasi-in situ XPS measurements of TiO₂−x during exposure to an H₂O atmosphere. (a) Time-resolved O 1s and (b) Ti 2p core-level spectra recorded during 30 min of H₂O exposure. The x-axis represents binding energy (eV), the y-axis represents exposure time (min), and the color scale represents signal intensity. The dashed vertical line in (a) indicates the characteristic binding-energy position of O²⁻. The dashed vertical lines in (b) indicate the characteristic binding-energy positions of Ti³⁺ and Ti⁴⁺, illustrating the time-dependent evolution of the surface chemical states during H₂O exposure. These measurements were performed using a cathodic-bias surrogate for ultrasound-generated charge carriers and should not be interpreted as a direct operando measurement under ultrasonic cavitation.

**Figure S10**. Reproducibility of the AIMD-derived proton-transfer free-energy profiles at the MXene/TiO₂ heterointerface. Three independent simulations (Run 1, Run 2, and Run 3) starting from different initial configurations yield consistent activation free energies of approximately 0.298, 0.318, and 0.251 eV at the transition state (TS), respectively. IS and FS denote the initial and final states. These AIMD profiles describe dynamic, solvent-assisted proton transfer at the defect-engineered heterointerface at 298 K, which is distinct from the static CI-NEB barrier calculated for H₂O dissociation on a pristine, defect-free TiO₂(101) surface in vacuum.

**Figure S11.** Antibacterial kinetics of MXene-based composites under 40 kHz ultrasound in different media. (a, b) Time-dependent survival of (a) *E. coli* and (b) *S. aureus* in pure water under various treatments: control (40 kHz ultrasound alone), 475 mg L⁻¹ Ti₃AlC₂, 475 mg L⁻¹ MXene/TiO₂, and HT100. (c, d) Survival of (c) *E. coli* and (d) *S. aureus* in blueberry juice under 40 kHz ultrasound with and without 475 mg L⁻¹ MXene/TiO₂. (e, f) Survival of (e) *E. coli* and (f) *S. aureus* in celery juice under the same treatment conditions as in c and d. In all kinetic panels (a-f), survival is expressed as log(N/N₀) over time (min). The MXene/TiO₂ composite demonstrates enhanced bactericidal activity across all media. (g) Schematic illustration of the performed antibacterial treatment in the three liquid media (water, blueberry juice, celery juice). All ultrasonic treatments were conducted in a thermostated bath with external circulating cooling at 25 ± 2 °C. The measured sample-temperature increase was maintained below 3 °C over 30 min, minimizing the contribution of bulk thermal effects to bacterial inactivation.

**Figure S12**. The concentration-dependent bactericidal kinetics of the MXene/TiO₂ composite against *E. coli* under 40 kHz ultrasound irradiation. Bacterial inactivation, expressed as log(N₀/N), is plotted over a treatment period of 30 minutes. Data points represent means of biological replicates, and lines are drawn as a visual guide. The composite exhibits a clear dose-response effect, where higher concentrations (▲ 425 mg L⁻¹ and ▼ 475 mg L⁻¹) achieve faster and more complete inactivation compared to lower concentrations (■ 250 mg L⁻¹ and ● 375 mg L⁻¹). The 475 mg L⁻¹ treatment demonstrates the most potent antibacterial activity.

**Figure S13**. Flow cytometry analysis of bacterial membrane integrity and ROS generation. Histograms show cell count distributions for *E. coli* (a, b) and *S. aureus* (c, d) after different treatments: control (red), 40 kHz ultrasound alone (blue), and 40 kHz ultrasound with MXene/TiO₂ (orange). (a, c) SYTO 9 staining reflects membrane integrity, with a left-shift in the 40 kHz/MXene/TiO₂ group indicating widespread membrane damage and cell death. (b, d) DCFH-DA staining detects intracellular ROS, with the 40 kHz/MXene/TiO₂ treatment showing a pronounced right-shift, consistent with pronounced oxidative stress associated with the composite catalyst.

**Figure S14**. Morphological characterization of bacterial cells before and after treatment.

**Figure S15**. Impact of Sonodynamic Treatment on Bioactive Compounds and Sensory Profiles in Fruit and Vegetable Juices. (a-c) Retention of bioactive compounds. Bar charts showing (a) total anthocyanin content (mg L⁻¹) and (b, c) vitamin C content (μg mL⁻¹) in juice samples inoculated with *E. coli* or *S. aureus*. Panels (b) and (c) represent distinct experimental sets, differing in the juice matrix used for treatment (blueberry vs. celery). Treatment groups are color-coded: Control (orange), 40 kHz (green), 40 kHz/MXene/TiO₂ (purple), and HT-100 (yellow). (d–f) Electronic-nose analysis of volatile profiles. Electronic-nose profiles illustrate the chemical fingerprint of treated juices. Panels (d) and (e) show radar plots of sensor responses for blueberry juice and celery juice, respectively, while (f) presents the principal component analysis (PCA) score distribution of celery juice samples, with ellipses indicating the clustering of different treatment groups. (g) Sensory attribute analysis. Radar chart depicting the multidimensional sensory profile of celery juice under different treatments. Each axis represents a specific sensory attribute (e.g., sourness, bitterness, astringency, richness, and aftertaste), and the colored lines indicate the sensory signature of each treatment group. In panels (a–c), data are presented as mean ± SD (n = 3). Treatments sharing no lowercase letters differ significantly within each bacterial species and juice matrix (p < 0.05).

**Figure S16**. Effects of different treatment systems on the survival and morphology of Caenorhabditis elegans. (a) Effects of water, blueberry juice, and celery juice at different exposure concentrations (1%, 2%, and 5%, v/v) on nematode survival after 24 h, which were used to determine the concentration for subsequent exposure experiments. (b) Based on the concentration-screening results, 2% (v/v) was selected to evaluate the effects of M9 control, water, blueberry juice, celery juice, and their corresponding MXene/TiO₂-treated systems on nematode survival after 24 h. (c) Representative images of nematodes before exposure (0 h) and after 24 h exposure. Scale bar: 50 μm. Data are presented as mean ± SD (n = 3). These results represent preliminary safety-related observations based on a short-term (24 h) exposure; definitive toxicological assessment requires chronic and mammalian studies.

**Figure S17.** Comparative life cycle assessment (LCA) of sonocatalytic (40 kHz/MXene/TiO₂) and high-pressure processing (HPP) for juice sterilization. (a) Cost breakdown (in %) for blueberry juice and celery juice. The stacked bars decompose the total cost into four categories: Raw Material, Preparation, Sterilization, and Packaging. The sonocatalytic process demonstrates a notably lower overall cost for both juices, primarily due to a significant reduction in the sterilization and preparation components. (b) Environmental impact analysis in terms of global warming potential (GWP, in kg CO₂ equivalent). The bars show the contribution from four life cycle stages: Energy, Material, Pre-processing, and Transportation. The 40 kHz/MXene/TiO₂ treatment exhibits a substantially lower GWP compared to HPP, with the energy consumption during the sterilization phase being the most prominent factor reduced.

**Figure S18.** Reusability of the MXene/TiO₂ sonosensitizer.

**Figure S19.** X-ray diffraction (XRD) patterns of pristine P25, MXene, and MXene/TiO₂, together with the corresponding samples after 40 kHz ultrasonic treatment for 30 and 60 min. The diffraction patterns are vertically offset for clarity.

**S2.0 Table**

**Table S1. Comparison of reported ultrasound-based juice sterilization methods and the present 40 kHz/MXene/TiO₂ system.**

| **Ultrasound equipment** | **Frequency** | **Power** | **Microorganism** | | | **Initial concentration** | **Juice type** | **Treatment time** | **Combined treatment** | **Inactivation efficiency** | **Reference** |
| --- | --- | --- | --- | --- | --- | --- | --- | --- | --- | --- | --- |
| Probe-type ultrasound system (UIP2000hdT, Hielscher Ultrasonics GmbH) | 20 kHz | 2000 W; total energy input of 40 W mL⁻¹ | TBC (total bacterial count); Y&M (yeasts and molds) | | | TBC (3.63 log CFU mL⁻¹); Y&M (3.20 log CFU mL⁻¹) | Freshly squeezed orange juice (Navel variety) | 20 min | High-pressure processing (450 MPa, 3 min) | ND（Not Detected） | [60] |
| Probe-type ultrasound system (Hielscher UP400S, 22 mm probe) | 24 kHz | 2155–2204  mW mL⁻¹ | *E. coli* ATCC 11775 | | | Not reported | Carrot juice | 10 min | Mild heat treatment (58 °C) | *E. coli* achieved the FDA-required 5-log reduction | [61] |
| Probe-type ultrasound system (ATPIO-1000D, Nanjing Xianou, China) | 20–25 kHz | 700 W; pulsed mode, 2 s on /3 s off | *E. coli*, TBC, Y&M | | | TBC: 4.25 log CFU mL⁻¹; *E. coli*: 3.04 log MPN mL⁻¹; yeasts: 2.40 log CFU mL⁻¹; molds: 0.73 log CFU mL⁻¹ | Grape juice (No. 8 Hutai cultivar) | 30 min | Mild heat treatment (55 °C) and nisin supplementation (200 ppm) | TBC reduced by 99.7%; *E. coli*: ND; Y&M: ND | [62] |
| Probe-type ultrasound system (UP200ST, Hielscher, Germany) | 26 kHz | 200 W; amplitude of 90 μm | *E. coli* K12 WT, *Listeria monocytogenes* EGDe | | | 10⁸ CFU mL⁻¹ | Pineapple juice | *E. coli*:255 s；*L. monocytogenes*：300 s | Mild heat treatment (59–62 °C) | *E. coli* K12 WT reduced by 5.21 log; *L. monocytogenes* WT reduced by 5.69 log | [63] |
| Ultrasonic bath (Elmasonic S 300H, Germany) | 37 kHz | 300 W | | | *Escherichia coli* PTCC 1789, *Salmonella Typhi* PTCC 1609 | 8.1 log CFU mL⁻¹ | Celery juice | 6 min | Heat treatment at 70 °C | *E. coli* PTCC 1789：ND *Salmonella* Typhi PTCC 1609：ND | [64] |
| Digital ultrasonic bath (Grant Instruments XUB 25, UK; capacity: 28 L) | 40 kHz | 400 W; acoustic energy density of 0.348 W cm⁻³ | | TBC; Y&M | | TBC: 5.32 log CFU mL⁻¹；Y&M: 4.51 log CFU mL⁻¹ | Hog plum (Spondias mombin L.) juice | 30 min | Heat treatment at 60 °C | TBC, Y&M：ND，≥5 log reduction | [65] |
| Digital ultrasonic bath (Grant Instruments XUB 25, UK; capacity: 25 L) | 40 kHz | 400 W; acoustic energy density of 0.348 W cm⁻³ | TBC; Y&M | | | TBC: 5.45 log CFU mL⁻¹；Y&M: 4.34 log CFU mL⁻¹ | African mango (Irvingia gabonensis) juice | 30 min | Heat treatment at 65 °C | TBC and Y&M were not detected, although a reduction in nutritional quality was observed | [66] |
| Probe-type ultrasound system (Q700, QSonica LLC, USA; 13 mm probe) | 20 kHz | 0.401–0.668 W, corresponding to 30–50 W cm⁻³ | *E. coli* ATCC 11775, *S. aureus* ATCC 12600 | | | 7 log CFU mL⁻¹ | Freshly squeezed orange juice (Citrus sinensis cv. Valencia) | 5 min | Photodynamic treatment using curcumin as the photosensitizer (100 μM) and blue LED irradiation for 3 h (462 ± 3 nm, 70 J cm⁻²) | *E. coli* and *S. aureus* were reduced by 4.26 and 2.35 log CFU mL⁻¹, respectively | [67] |
| Probe-type ultrasound system (Xinyi-950N, Xinyi Inc., Hangzhou, China; 6 mm probe) | 20 kHz | 700 W | *E. coli* CGMCC1.2463, *S. aureus* CICC 21601 | | | 10⁶ CFU mL⁻¹ | Cloudy NFC apple juice prepared from Fuji apples | 10 min | Phloretin supplementation (300 μg mL⁻¹) | *E. coli* and *S. aureus* were reduced by 6.5 and 6.6 log, respectively | [68] |
| Ultrasonic bath (Elmasonic Easy 30H, Germany) | 37 kHz | 500 W at 70% output power | TBC; Y&M | | | TBC：5.41 log CFU mL⁻¹；Y&M ：4.35 log CFU mL⁻¹ | Sugarcane juice (Saccharum officinarum, cultivar CP72–2086) | 15 min | Heat treatment at 65 °C combined with natural preservatives | TBC and Y&M were reduced by 3.76 and 3.11 log, respectively | [69] |
| Probe-type ultrasound system (Vibra-Cell VC505, Sonics & Materials, USA; 19 mm probe) | 20 kHz | 500 W | *E. coli* ATCC 25922 ；*Saccharomyces cerevisiae* ATCC 2366 | | | *E. coli* ATCC 25922, S. cerevisiae ATCC 2366 ：10⁶–10⁷ CFU mL⁻¹；TBC：2 log CFU mL⁻¹，Y&M：1.36 log CFU mL⁻¹ | Clarified pomegranate juice (Punica granatum L., cv. Hicaz) | 30 min | None | *E. coli* and *S. cerevisiae* were reduced by 6.64 and 1.36 log, respectively; TBC and Y&M were not detected | [70] |
| Ultrasonic system (AK-040SD; temperature controlled using circulating cooling water) | 40 kHz | 360 W | *E. coli* ATCC 25922, *S. aureus* ATCC 2921, Penicillium italicum, and P. expansum | | | *E. coli*: 2 × 10⁷ CFU mL⁻¹; *S. aureus*: 2 × 10⁹ CFU mL⁻¹; fungal spore suspension: 10⁴–10⁵ spores mL⁻¹ | Shine Muscat grape | 20 min | Xenon-lamp irradiation (200 mW cm⁻²) combined with sonodynamic treatment using HB@ZIF-8 nanoparticles (ZHB) | *S. aureus* and *E. coli* were reduced by approximately 9 and 7 log CFU mL⁻¹, respectively; P. italicum was completely inhibited | [71] |
| Ultrasonic bath (WN-020S) | 40 kHz | 120 W | *E. coli* ATCC 25922 and *S. aureus* CMCC(B) 26003 | | | 1×10^7^ CFU mL⁻¹ | Blueberry juice and celery juice | 30 min（Blueberry juice）and 60 min（celery juice） | 475 mg L⁻¹ MXene/TiO₂ | Both *E. coli* and *S. aureus* were reduced by more than 3 log | This work |

**Table S2. Total Ti concentrations in water, blueberry juice, and celery juice following different treatments.**

| **Sample Matrix** | **Treatment** | **Total Ti Concentration (mg L⁻¹)** |
| --- | --- | --- |
| Water | Control | 0.15109 ± 0.00012 |
|  | 40 kHz | 0.29281 ± 0.01090 |
|  | 40 kHz/MXene/TiO₂ | 0.45746 ± 0.02168 |
| Blueberry juice | Control | 0.05578 ± 0.00025 |
|  | 40 kHz | 0.09853 ± 0.00124 |
|  | 40 kHz/MXene/TiO₂ | 0.27826 ± 0.00617 |
| Celery juice | Control | 0.08052 ± 0.00289 |
|  | 40 kHz | 0.14504 ± 0.00193 |
|  | 40 kHz/MXene/TiO₂ | 0.22093 ± 0.00192 |

**Note:** Total Ti concentrations are reported as upper-bound estimates; background contributions from non-catalyst sources are noted. These data are preliminary safety-related observations, not toxicological validation.

**S3.0 Text**

**Text S1. 2D passive cavitation imaging (PCI)**

The measurements were performed in the aforementioned ultrasonic cleaner operating at 40 kHz. A rectangular container was fixed at the bottom of the cleaner. The container consisted of an ABS frame with dimensions of 58 mm × 33 mm × 33 mm and an optically and acoustically transparent membrane film (3L film, 3L Medical Products Group, China). Inlet and outlet ports were installed on the left and right sides of the container, respectively, to introduce liquids containing different materials. A 128-element linear array was positioned approximately 1 cm above the upper surface of the container to passively receive scattered signals generated by cavitation. Both the container and the linear array were immersed in water. The linear array was connected to a diagnostic ultrasound platform for real-time monitoring of cavitation intensity, and the raw radiofrequency data were synchronously stored on a PC. During measurement, the ultrasonic cleaner was activated, and raw radiofrequency signals were continuously recorded for 2 s.

Post-processing of the radiofrequency signals mainly consisted of two steps: beamforming and filtering. First, the minimum variance delay-and-sum (MV-DAS) algorithm was used to reconstruct the data, allowing the cavitation signal distribution in a two-dimensional plane to be obtained while suppressing noise. Subsequently, a high-pass filter with a cutoff frequency of 40 kHz was applied to remove the fundamental frequency component. Because cavitation activity is mainly reflected by enhanced high-frequency components, the intensity of the filtered signals can be used to represent the relative strength of cavitation activity.

For quantitative comparison, cavitation intensity was calculated as the squared norm of the filtered signal,
𝑰=|| Filtered Signal ||^𝟐^. The spatially averaged cavitation intensity was obtained by averaging all sampling points within the same imaging region for each frame. Values obtained at 0, 0.72, 1.44, and 2.17 s were subsequently averaged to obtain the time-averaged cavitation intensity. The same beamforming, filtering, spatial-sampling, intensity-calculation, and averaging procedures were applied to the water, MAX, P25, and MXene/TiO₂ groups, and all PCI maps were displayed using the same intensity scale of 0–2000 a.u.

**Text S2. Fluid-Solid Coupling Model for Cavitation Bubbles near a Solid Interface**

Numerical simulations were performed using the finite-element software COMSOL Multiphysics to illustrate the plausible radius evolution and acoustic streaming distribution, and to model the stress-transfer process of a single bubble near the MXene/TiO₂ heterointerface under ultrasonic excitation. Unlike the aluminum foil erosion assay, this model focuses on the mechanical response of the catalyst particles themselves, treating the interface as a rigid boundary with material properties representative of the composite nanostructure. This single-bubble model represents a simplified description of the cavitation field and does not capture collective multi-bubble interactions under experimental sonication conditions. Accordingly, the simulation results serve as mechanistic illustration of the proposed energy-focusing hypothesis rather than as direct experimental evidence.

After bubble formation, its oscillatory behavior near a rigid boundary can be described by the Rayleigh–Plesset equation:

$$\text{R}\frac{\text{d}^{\text{2}}\text{R}}{\text{d}\text{t}^{\text{2}}}\text{+}\frac{\text{3}}{\text{2}}\text{ρ}\left( \frac{\text{dR}}{\text{dt}} \right)^{\text{2}}\text{=}\text{P}_{\text{g}}\text{-}\text{P}_{\text{0}}\text{-}\text{P}\text{(}\text{t}\text{)-}\frac{\text{4}\text{μ}}{\text{R}}\frac{\text{dR}}{\text{dt}}\text{-}\frac{\text{2}\text{σ}}{\text{R}}\text{,}$$

where $\text{R}$ is the microbubble radius, $\text{ρ}$ is the liquid density, $\text{P}\text{(}\text{t}\text{)}$ is the external pressure induced by the driving acoustic field, $\text{μ}$ is the dynamic viscosity of the liquid, and $\text{σ}\text{ }$is the surface tension at the gas-liquid interface.

Furthermore, bubble oscillation induces unsteady motion in the surrounding fluid, and the resulting flow behavior can be described by the continuity equation and the Navier-Stokes equation:

$$\frac{\text{∂}\text{ρ}}{\text{∂}\text{t}}\text{+∇⋅(}\text{ρ}\text{v}\text{)=0,}$$

$$\text{ρ}\left( \frac{\text{∂}\text{v}}{\text{∂}\text{t}}\text{+}\text{v}\text{⋅∇}\text{v} \right)\text{=∇⋅}\left[ \text{-}\text{P}\text{I}\text{+}\text{μ}\left( \text{∇}\text{v}\text{+(∇}\text{v}\text{)}^{\text{T}} \right) \right]\text{.}$$

Where $\text{v}$ is the liquid velocity vector, $\text{P}$is the pressure in the liquid, and $\text{I}$ is the identity tensor.

The von Mises stress generated in the material was calculated from the solid displacement u, and the displacement field satisfies the solid dynamics equation:

$$\text{ρ}_{\text{s}}\frac{\text{∂}^{\text{2}}\text{u}}{\text{∂}\text{t}^{\text{2}}}\text{-∇⋅}\text{σ}_{\text{s}}\text{=0,}$$

Where $\text{ρ}_{\text{s}}$is the solid density and $\text{σ}_{\text{s}}$ is the solid stress tensor. In this model, the external load applied to the solid originates from the pressure generated by fluid flow, and the following boundary condition must be satisfied at the solid-liquid interface:

$$\text{σ}_{\text{s}}\text{⋅}\text{n}\text{=}\text{σ}_{\text{f}}\text{⋅}\text{n}\text{,}$$

The specific mechanical parameters used for the MXene/TiO₂ composite domain were carefully selected to reflect the intrinsic properties of the nanostructured catalyst. Density (ρs): Set to 4200 kg m⁻³, representing a weighted average consistent with the densities of anatase TiO₂ (~3900 kg m⁻³) and Ti₃C₂Tₓ MXene (~4300 kg m⁻³). Young’s Modulus: Set to 230 GPa. While bulk anatase TiO₂ typically exhibits a modulus of 250–300 GPa, the value was moderately adjusted to account for the presence of oxygen vacancies and the layered, slightly compliant nature of the MXene phase within the composite, which reduces the effective stiffness compared to pristine single-crystal TiO₂.

Poisson's Ratio: Set to 0.27, typical for ceramic oxides. Thickness: Set to 200 nm, representative of the hydrodynamic radius of the aggregated catalyst particles used in the experiments. The bubble surface tension was set to 0.072 N m⁻¹ (water–air interface). The initial bubble radius was set to 50 μm—a value close to the resonant size for 40 kHz ultrasound in water—to ensure physically meaningful cavitation dynamics. In selected sensitivity analyses, a larger pre-existing nucleus (200 μm radius) was also tested to evaluate the effect of non-resonant nuclei. The distance from the bubble center to the upper surface of the material was set to 400 μm. The ultrasonic frequency was set to 40 kHz, and the acoustic pressure amplitude was 0.5 MPa.

**Text S3. Description of Time-Resolved Cavitation Imaging**

In addition to the time points discussed below(0, 0.72, and 1.44 s), data at 2.17 s were also analyzedand showed consistent trends (see Text S1 for methodology). During cavitation, bubbles undergo violent expansion and contraction and may even collapse, exhibiting highly unstable dynamic behavior. Therefore, to verify that the strong cavitation phenomenon reported in the main text was not an accidental event, cavitation imaging results for each experimental group were calculated at three representative time points: t = 0 s, 0.72 s, and 1.44 s. The control group showed relatively weak cavitation activity at all three time points, possibly due to the limited number of available cavitation nuclei in water. In the MAX and P25 groups, scattered red signal spots appeared in certain regions, and their spatial positions changed over time; however, the overall cavitation intensity was still markedly higher than that of the control group. In contrast, the MXene/TiO₂ group exhibited extensive and intense cavitation activity at multiple time points, consistent with the composite producing more intense cavitation activity under ultrasonic irradiation.

**S4.0 Statistical analysis**

**Table S3. Bacterial inactivation at the final treatment time under different treatments.**

| **Figure** | **Matrix** | **Microorganism** | **Time** | **Control** | **Ti₃AlC₂** | **475 mg L⁻¹ MXene/TiO₂** | **HT100** |
| --- | --- | --- | --- | --- | --- | --- | --- |
| S11a | Water | *E. coli* | 30 min | 0.713 ± 0.038ᶜ | 1.027 ± 0.110ᵇ | 3.242 ± 0.084ᵃ | 3.215 ± 0.079ᵃ |
| S11b |  | *S. aureus* |  | 0.663 ± 0.047ᶜ | 1.270 ± 0.050ᵇ | 3.237 ± 0.055ᵃ | 3.250 ± 0.050ᵃ |
| S11c | Blueberry juice | *E. coli* |  | 1.055 ± 0.058ᵇ | — | 3.283 ± 0.047ᵃ | 3.283 ± 0.047ᵃ |
| S11d |  | *S. aureus* |  | 1.047 ± 0.055ᵇ | — | 3.270 ± 0.030ᵃ | 3.283 ± 0.047ᵃ |
| S11e | Celery juice | *E. coli* | 60 min | 1.889 ± 0.052ᵇ | — | 3.192 ± 0.051ᵃ | 3.175 ± 0.067ᵃ |
| S11f |  | *S. aureus* |  | 2.027 ± 0.064ᵇ | — | 3.360 ± 0.053ᵃ | 3.360 ± 0.053ᵃ |

Data are presented as mean ± SD (n = 3). Lowercase superscript letters indicate statistical groupings among treatments within the same row and are assigned in descending order of the mean value (a represents the group with the highest mean, followed by b and c). Values carrying different letters are significantly different from each other (p < 0.05), whereas values carrying the same letter are not significantly different. If two groups share at least one letter (e.g., ab), no significant difference exists between them. Statistical significance was assessed by two-way ANOVA followed by Tukey’s multiple-comparison test.

**S5.0 Addressing Spatial-Resolution Limitations and Physical-Effect Ambiguities in Electrochemical Transients**

This interpretation should be regarded as a mechanistic assignment supported by indirect evidence. Individual cavitation events at 40 kHz occur on microsecond timescales (acoustic period ≈ 25 μs) and therefore cannot be resolved by millisecond-scale sampling. The recorded signals are further shaped by two cascaded low-pass processes: (i) the electrochemical cell time constant τ = R_u_·C_d_l, which at macroelectrode dimensions is on the millisecond scale; and (ii) spatial ensemble averaging over the entire electrode area, where large numbers of microsecond-scale bubble events superimpose statistically. Accordingly, this work interprets the chronoamperometric transients not as direct records of single interfacial charge-transfer events, but as the ensemble envelope of cavitation-enhanced interfacial processes, including mass-transport (surface-renewal) enhancement by acoustic streaming. This assignment is quantitatively consistent with our existing data: the uncompensated resistance obtained from the high-frequency intercept of our EIS spectra (Figure S5a), combined with the double-layer capacitance derived from the same measurements, yields a cell time constant that falls within the range of the observed transient relaxation behavior. In addition, the insulating SiO₂ control (Figure S5c) shows no discernible response under otherwise identical conditions, indicating that the response requires an electronically conductive interface rather than arising purely from bulk or capacitive effects of the electrolyte under ultrasound.

**Table S4. Electronic-nose sensor responses of blueberry and celery juices under different treatments**

| **Sensor** | **Blueberry juice (Figure S15d)** | | | | **Celery juice (Figure S15e)** | | | |
| --- | --- | --- | --- | --- | --- | --- | --- | --- |
|  | **Control** | **40 kHz** | **MXene/TiO₂** | **40 kHz/MXene/TiO₂** | **Control** | **40 kHz** | **MXene/TiO₂** | **40 kHz/MXene/TiO₂** |
| W1C | 0.9980 ± 0.0414ᵃ | 0.9027 ± 0.0654ᵇ | 1.0068 ± 0.0198ᵃ | 0.9527 ± 0.0212ᵃᵇ | 0.8555 ± 0.0198ᵇ | 0.8523 ± 0.0099ᵇ | 0.8673 ± 0.0030ᵃᵇ | 0.8821 ± 0.0185ᵃ |
| W5S | 1.3592 ± 0.0805ᵇ | 1.7027 ± 0.0936ᵃ | 1.3503 ± 0.0717ᵇ | 1.6228 ± 0.0308ᵃ | 1.7223 ± 0.0329ᵇᶜ | 1.7589 ± 0.0035ᵃ | 1.7061 ± 0.0065ᶜ | 1.7448 ± 0.0066ᵃᵇ |
| W3C | 0.9983 ± 0.0297ᵃ | 0.9201 ± 0.0435ᵇ | 1.0067 ± 0.0160ᵃ | 0.9558 ± 0.0139ᵇ | 0.8583 ± 0.0060ᵃ | 0.8541 ± 0.0018ᵃ | 0.8556 ± 0.0011ᵃ | 0.8632 ± 0.0172ᵃ |
| W6S | 1.0625 ± 0.0155ᵇ | 1.0778 ± 0.0093ᵃ | 1.0383 ± 0.0089ᶜ | 1.0607 ± 0.0032ᵇ | 1.0960 ± 0.0019ᶜ | 1.1058 ± 0.0021ᵃ | 1.1019 ± 0.0005ᵇ | 1.1007 ± 0.0005ᵇ |
| W5C | 1.0021 ± 0.0267ᵃ | 0.9310 ± 0.0394ᵇ | 1.0097 ± 0.0139ᵃ | 0.9625 ± 0.0118ᵇ | 0.8693 ± 0.0054ᵃᵇ | 0.8666 ± 0.0009ᵇ | 0.8652 ± 0.0015ᵇ | 0.8781 ± 0.0126ᵃ |
| W1S | 1.0788 ± 0.1502ᵇ | 1.4849 ± 0.2396ᵃ | 1.0335 ± 0.0856ᵇ | 1.2998 ± 0.0770ᵃ | 1.8291 ± 0.0568ᵃ | 1.8991 ± 0.0286ᵃ | 1.8713 ± 0.0077ᵃ | 1.8282 ± 0.1195ᵃ |
| W1W | 1.3286 ± 0.1198ᵇ | 1.6431 ± 0.1289ᵃ | 1.3213 ± 0.0808ᵇ | 1.5421 ± 0.0520ᵃ | 1.6534 ± 0.0070ᵃ | 1.6509 ± 0.0119ᵃ | 1.6055 ± 0.0076ᵇ | 1.5587 ± 0.0460ᶜ |
| W2S | 1.0021 ± 0.0029ᵃ | 1.0040 ± 0.0047ᵃ | 1.0015 ± 0.0038ᵃ | 1.0053 ± 0.0053ᵃ | 1.0081 ± 0.0038ᵃ | 1.0010 ± 0.0055ᵃᵇ | 0.9963 ± 0.0094ᵇ | 1.0034 ± 0.0059ᵃᵇ |
| W2W | 1.3765 ± 0.1068ᵇ | 1.6002 ± 0.0931ᵃ | 1.3592 ± 0.0707ᵇ | 1.5153 ± 0.0387ᵃ | 1.5582 ± 0.0126ᵃ | 1.5246 ± 0.0044ᵇ | 1.4972 ± 0.0043ᶜ | 1.4462 ± 0.0257ᵈ |
| W3S | 1.0360 ± 0.0103ᵃ | 1.0352 ± 0.0063ᵃ | 1.0231 ± 0.0082ᵇ | 1.0252 ± 0.0073ᵃᵇ | 1.0342 ± 0.0100ᵇ | 1.0112 ± 0.0053ᶜ | 1.0123 ± 0.0035ᶜ | 1.0470 ± 0.0088ᵃ |

Data are presented as mean ± SD (n = 5). Lowercase superscript letters (a–d) denote the statistical grouping of treatments for each sensor within the same juice and are assigned in descending order of the mean value, with “a” representing the group with the highest mean, followed by b, c, and d. Values carrying different letters are significantly different from each other (p < 0.05), whereas values carrying the same letter are not significantly different. Values sharing at least one letter (e.g., ab) are also not significantly different. Statistical significance was assessed by one-way ANOVA followed by Duncan’s multiple range test. The data for blueberry juice and celery juice correspond to Figures S15d and S15e, respectively.

**Table S5. Electronic-tongue responses of blueberry and celery juices under different treatments**

| **Taste attribute** | **Blueberry juice (Figure 6b)** | | | | **Celery juice (Figure S15g)** | | | |
| --- | --- | --- | --- | --- | --- | --- | --- | --- |
|  | **Control** | **40 kHz** | **MXene/TiO₂** | **40 kHz/MXene/TiO₂** | **Control** | **40 kHz** | **MXene/TiO₂** | **40 kHz/MXene/TiO₂** |
| Sourness | 11.080 ± 0.000ᵃ | 11.123 ± 0.049ᵃ | 11.167 ± 0.042ᵃ | 11.093 ± 0.050ᵃ | −36.900 ± 0.056ᶜ | −35.810 ± 0.030ᵇ | −35.710 ± 0.085ᵃᵇ | −35.640 ± 0.026ᵃ |
| Bitterness | −0.370 ± 0.000ᵇ | −0.570 ± 0.026ᶜ | −0.167 ± 0.049ᵃ | −0.627 ± 0.006ᵈ | 7.240 ± 0.111ᶜ | 7.430 ± 0.044ᵇ | 7.920 ± 0.056ᵃ | 7.960 ± 0.030ᵃ |
| Astringency | 1.430 ± 0.000ᵃ | 1.303 ± 0.040ᵇ | 1.210 ± 0.035ᶜ | 1.250 ± 0.010ᶜ | 0.290 ± 0.010ᵈ | 0.810 ± 0.010ᵃ | 0.460 ± 0.010ᶜ | 0.610 ± 0.010ᵇ |
| Aftertaste-B | 0.290 ± 0.000ᵇ | 0.073 ± 0.057ᶜ | 0.583 ± 0.067ᵃ | 0.103 ± 0.055ᶜ | −0.070 ± 0.010ᶜ | 0.010 ± 0.010ᵇ | −0.070 ± 0.010ᶜ | 0.380 ± 0.010ᵃ |
| Aftertaste-A | 0.410 ± 0.000ᵃ | 0.387 ± 0.029ᵃ | 0.407 ± 0.035ᵃ | 0.403 ± 0.015ᵃ | 0.120 ± 0.010ᶜ | 0.300 ± 0.010ᵃ | 0.150 ± 0.010ᵇ | 0.160 ± 0.010ᵇ |
| Umami | −6.870 ± 0.000ᵃ | −6.900 ± 0.010ᵃ | −6.890 ± 0.010ᵃ | −6.893 ± 0.021ᵃ | 3.630 ± 0.098ᵃ | 3.570 ± 0.035ᵃ | 2.680 ± 0.030ᶜ | 3.340 ± 0.030ᵇ |
| Richness | −0.110 ± 0.000ᵃ | −0.107 ± 0.125ᵃ | −0.087 ± 0.129ᵃ | −0.113 ± 0.095ᵃ | −0.080 ± 0.010ᶜ | 0.050 ± 0.017ᵃ | 0.010 ± 0.010ᵇ | 0.030 ± 0.010ᵃᵇ |
| Saltiness | −11.750 ± 0.000ᵃᵇ | −11.843 ± 0.038ᶜ | −11.800 ± 0.040ᵇᶜ | −11.730 ± 0.010ᵃ | 6.020 ± 0.030ᵃ | 5.960 ± 0.089ᵃ | 4.620 ± 0.036ᶜ | 5.600 ± 0.044ᵇ |

Data are presented as mean ± SD (n = 3). Lowercase superscript letters (a–d) indicate the statistical grouping of treatments for each taste attribute within the same juice and are assigned in descending order of the mean value, with “a” representing the group with the highest mean, followed by b, c, and d. Values carrying different letters are significantly different from each other (p < 0.05), whereas values carrying the same letter are not significantly different. Values sharing at least one letter (e.g., ab) are also not significantly different. Statistical significance was assessed by one-way ANOVA followed by Duncan’s multiple range test. The blueberry juice and celery juice data correspond to Figure 6b and Figure S15g, respectively.

**S6.0 Supporting Information References**

**60.** B.-S. Chu, M. A. Lemos, S. Blidi, et al., “Characterisation of Fresh-Like Orange Juice Prepared by Ultrasound Treatment Followed by High-Pressure Processing under Industrial Setting,” *LWT* 228 (2025): 118019, https://doi.org/10.1016/j.lwt.2025.118019.

**61.** H. E. Martínez-Flores, M. G. Garnica-Romo, D. Bermúdez-Aguirre, P. R. Pokhrel, and G. V. Barbosa-Cánovas, “Physico-Chemical Parameters, Bioactive Compounds and Microbial Quality of Thermo-Sonicated Carrot Juice during Storage,” *Food Chemistry* 172 (2015): 650–656, https://doi.org/10.1016/j.foodchem.2014.09.072.

**62.** T. Ma, J. Wang, L. Wang, et al., “Ultrasound-Combined Sterilization Technology: An Effective Sterilization Technique Ensuring the Microbial Safety of Grape Juice and Significantly Improving Its Quality,” *Foods* 9 (2020): 1512, https://doi.org/10.3390/foods9101512.

**63.** E. T. Okafor, F. Pavli, C. O’Bryne, and V. Valdramidis, “Insights on the Antimicrobial Mechanisms of Action of High Power Ultrasound,” *Innovative Food Science & Emerging Technologies* 104 (2025): 104135, https://doi.org/10.1016/j.ifset.2025.104135.

**64.** S. M. B. Hashemi, R. Roohi, and E. Abedi, “Thermodynamics, Kinetics, and Computational Fluid Dynamics Modeling of *Escherichia coli* and *Salmonella Typhi* Inactivation during the Thermosonication Process of Celery Juice,” *Ultrasonics Sonochemistry* 104 (2024): 106820, https://doi.org/10.1016/j.ultsonch.2024.106820.

**65.** A. O. Oladunjoye, F. O. Adeboyejo, T. A. Okekunbi, and O. R. Aderibigbe, “Effect of Thermosonication on Quality Attributes of Hog Plum (*Spondias mombin* L.) Juice,” *Ultrasonics Sonochemistry* 70 (2021): 105316, https://doi.org/10.1016/j.ultsonch.2020.105316.

**66.** A. O. Oladunjoye and E. U. Awani-Aguma, “Effect of Thermosonication on Physicochemical, Bioactive, Sensory and Microbiological Qualities of African Mango Fruit (*Irvingia gabonensis*) Juice,” *Measurement: Food* 11 (2023): 100103, https://doi.org/10.1016/j.meafoo.2023.100103.

**67.** M. L. Bhavya and H. U. Hebbar, “Sono-Photodynamic Inactivation of *Escherichia coli* and *Staphylococcus aureus* in Orange Juice,” *Ultrasonics Sonochemistry* 57 (2019): 108–115, https://doi.org/10.1016/j.ultsonch.2019.05.002.

**68.** L. Wei, C. Shi, C. Y. Hu, et al., “Inactivation Effect of Phloretin with Ultrasound on *Staphylococcus aureus* and *Escherichia coli* Extended the Shelf-Life of Cloudy Apple Juice,” *Food Bioscience* 68 (2025): 106498, https://doi.org/10.1016/j.fbio.2025.106498.

**69.** T. Bibi, A. Liaqat, T. Mehmood, et al., “Combined Effect of Thermosonication and Natural Preservatives on the Quality of Sugarcane Juice,” *Food Chemistry: X* 29 (2025): 102861, https://doi.org/10.1016/j.fochx.2025.102861.

**70.** C. U. Pala, N. N. D. Zorba, and G. Ozcan, “Microbial Inactivation and Physicochemical Properties of Ultrasound-Processed Pomegranate Juice,” *Journal of Food Protection* 78 (2015): 531–539, https://doi.org/10.4315/0362-028X.JFP-14-290.

**71.** L. Ding, Y. Xie, X. Bai, et al., “Sonodynamic Interfacial-Assisted Photosensitizer Disrupts Lipid Bilayer Order to Eradicate Gram-Negative Bacteria Using HB@ZIF-8 Nanoparticles,” *Food Chemistry* 495 (2025): 146316, https://doi.org/10.1016/j.foodchem.2025.146316.
